# Wavelet-based multifractal analysis of dynamic infrared thermograms to assist in early breast cancer diagnosis

**DOI:** 10.3389/fphys.2014.00176

**Published:** 2014-05-08

**Authors:** Evgeniya Gerasimova, Benjamin Audit, Stephane G. Roux, André Khalil, Olga Gileva, Françoise Argoul, Oleg Naimark, Alain Arneodo

**Affiliations:** ^1^Laboratory of Physical Foundation of Strength, Institute of Continuous Media Mechanics UB RASPerm, Russia; ^2^Laboratoire de Physique, ENS de Lyon, CNRS, UMR 5672, Université de LyonLyon, France; ^3^Department of Mathematics and Statistics, University of MaineOrono, ME, USA; ^4^Department of Therapeutic and Propedeutic Dentistry, Perm State Academy of MedicinePerm, Russia

**Keywords:** breast cancer, infrared thermography, wavelet transform modulus maxima method

## Abstract

Breast cancer is the most common type of cancer among women and despite recent advances in the medical field, there are still some inherent limitations in the currently used screening techniques. The radiological interpretation of screening X-ray mammograms often leads to over-diagnosis and, as a consequence, to unnecessary traumatic and painful biopsies. Here we propose a computer-aided multifractal analysis of dynamic infrared (IR) imaging as an efficient method for identifying women with risk of breast cancer. Using a wavelet-based multi-scale method to analyze the temporal fluctuations of breast skin temperature collected from a panel of patients with diagnosed breast cancer and some female volunteers with healthy breasts, we show that the multifractal complexity of temperature fluctuations observed in healthy breasts is lost in mammary glands with malignant tumor. Besides potential clinical impact, these results open new perspectives in the investigation of physiological changes that may precede anatomical alterations in breast cancer development.

## 1. Introduction

It is widely recognized that early diagnosis is the key to breast cancer survival. X-ray mammography (Nass et al., 2000; Bronzino, 2006), the golden standard for breast cancer screening detection, has a rather high false-positive rating and is not always effective in detecting cancer in young women who generally have dense breast tissue (Jorgensen and Gotzsche, 2009; Tamini et al., 2009) and this despite the increasing use of computer-aided detection/diagnosis methods (Fenton et al., 2011). Biopsy is indeed the only conclusive diagnostic test for breast cancer, but the number of unnecessary biopsies is still too high (Vinitha Sree et al., 2009). Since the original observation by Lawson (1956) that skin temperature over a malignant tumor is higher than its neighbourhood, possibly resulting from abnormal increase of metabolic activity and vascular circulation in the tissues beneath (Yahara et al., 2003; Bronzino, 2006), IR thermography has been considered as a promising non-invasive screening method of breast cancer (Ng, 2009). However the suitability of static IR imaging for routine screening has been severely questioned (Head and Elliott, 2002; Bronzino, 2006), because of insufficient sensitivity for detection of deep lesions and limited knowledge of the relationship between surface temperature distributions and thermal diseases. Renewed interest in dynamic IR imaging (Etehadtavakol and Ng, 2013) comes from the rapid development of new digital IR thermography cameras with higher temperature resolution (0.08°C or better) and faster frame rate (70 Hz) (Joro et al., 2008a), combined with increasing knowledge of tumor angiogenesis including nitric oxide production of the cancer tissue causing local disturbances in vasomotor (automatic nervous control of smooth muscles forcing blood through capillaries) and cardiogenic phenomena as compared to normal tissues (Thomsen and Miles, 1998; Anbar et al., 2001).

The basis for diagnostic application of dynamic IR imaging is the detection of intensity variations in temperature rhythms generated by the cardiogenic (1–1.5 Hz) and vasomotor (0.1–0.2 Hz) frequencies (Button et al., 2004; Joro et al., 2008b). In this study we show that beyond intensity differences in these rhythms between normal and tumor breast tissues, the complexity of temperature fluctuations about these physiological perfusion oscillations is qualitatively different. Using a wavelet-based multi-scale analysis (Muzy et al., 1991, 1994; Arneodo et al., 1995) of temperature fluctuations, we propose to characterize the multifractal properties of these temperature time-series as an effective discriminating method for early screening procedures to identify women with high risk of breast cancer.

## 2. Methods of analysis

### 2.1. The wavelet transform

The wavelet transform (WT) is a mathematical microscope (Muzy et al., 1991, 1994; Arneodo et al., 1995) that is well suited for the analysis of complex non-stationary time-series such as those found in physiological systems (Ivanov et al., 1999; Goldberger et al., 2002), thanks to its ability to filter out low-frequency trends in the analyzed signal Σ (Materials and Methods). The WT is a space (or time in our study)-scale analysis which consists in expanding signals in terms of wavelets which are constructed from a single function, the “analyzing wavelet” ψ, by means of translations and dilations. The WT of a real-valued function Σ is defined as (Mallat, 1998):

(1) $$W_{\psi }[\Sigma ](t_{0},\;a)=\frac{1}{a}\int_{-\infty }^{+\infty }\Sigma (t)\psi \left(\frac{t-t_{0}}{a}\right)dt,$$

where *t*_0_ is the time parameter and *a* (> 0) the scale parameter. By choosing a wavelet ψ whose *n* + 1 first moments are zero [*∫* *t*^*n*^ ψ (*t*)*dt* = 0, 0 ≤ *m* ≤ *n*] (Supplementary Figure 1), one makes the WT microscope blind to order-*n* polynomial behavior, a prerequisite for multifractal fluctuations analysis (Muzy et al., 1991, 1994; Arneodo et al., 1995). Indeed this mathematical microscope can be seen as a singularity scanner. By increasing magnification (decreasing the scale parameter *a* → 0^+^) around a given point *t*, finer and finer details of Σ can be revealed and quantified via the estimate of the so-called Holder exponent *h*(*t*) (Muzy et al., 1991, 1994; Arneodo et al., 1995).

### 2.2. The wavelet transform modulus maxima method

The WT modulus maxima (WTMM) method was originally developed to generalize box-counting techniques (Arneodo et al., 1987) and to remedy for the limitations of the structure functions method to perform multifractal analysis of one-dimensional (1D) velocity signal in fully developed turbulence (Muzy et al., 1991, 1994; Arneodo et al., 1995). It has proved very efficient to estimate scaling exponents and multifractal spectra (Muzy et al., 1994; Delour et al., 2001; Audit et al., 2002). This method has been generalized in 2D for the multifractal analysis of rough surfaces (Arneodo et al., 2003) and then for the analysis of 3D scalar and vector fields (Kestener and Arneodo, 2004; Arneodo et al., 2008). It has been successfully applied in various areas of fundamental research (Arneodo et al., 1998a,b, 2002, 2003, 2008; Khalil et al., 2006; Roland et al., 2009; Roux et al., 2009; Arneodo et al., 2011). In the context of the present study, the 1D WTMM method has proved very efficient at discriminating between healthy and sick heart beat dynamics (Ivanov et al., 1999, 2001; Goldberger et al., 2002), whereas the 2D WTMM method can be used to detect microcalcifications and has great potential to assist in cancer diagnosis from digitized mammograms (Kestener et al., 2001; Arneodo et al., 2003).

The WT modulus maxima (WTMM) method (Muzy et al., 1991, 1994; Arneodo et al., 1995) consists in computing the WT skeleton defined, at each fixed scale *a*, by the local maxima 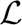
(*a*) of the WT modulus |*W*(*t, a*)|. These WTMM are disposed on curves connected across scales called maxima lines *l*_*t*_ (Supplementary Figure 2), along which the WTMM behave as *a*^*h*(*t*)^, where *h*(*t*) is the Hölder exponent (Muzy et al., 1991, 1994; Arneodo et al., 1995) characterizing the singularity of Σ at time *t*. The multifractal formalism amounts to characterize the relative contributions of each Hölder exponent value via the estimate of the *D*(*h*) singularity spectrum defined as the fractal dimension of the set of points *t* where *h*(*t*) = *h*. This spectrum can be obtained by investigating the scaling behavior of partition functions defined in terms of wavelet coefficients:

(2) $$Z(q,\;a)=\sum_{l\in ℒ(a)}|W(t,\;a){|}^{q}\sim a^{\tau (q)},$$

where *q* ϵ ℝ. Then from the scaling function τ(*q*), *D*(*h*) is obtained by a Legendre transform (Muzy et al., 1991, 1994; Arneodo et al., 1995):

(3) $$D(h)=\underset{q}{\min}[qh-\tau (q)].$$

As originally pointed out in Muzy et al. (1994); Arneodo et al. (1995), one can avoid some practical difficulties that occur when directly performing the Legendre transform of τ(*q*), by computing the following expectation values:

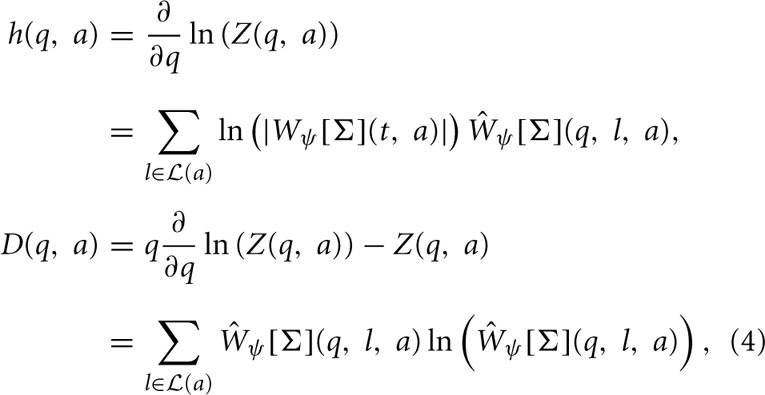



where *Ŵ*_*ψ*_[Σ](*q, l, a*) = $\frac{|W_{\psi }[\Sigma ](t,\;a){|}^{q}}{Z(q,\;a)}$ is the equivalent of Bolzmann weight in the analogy that links the multifractal formalism to thermodynamics (Arneodo et al., 1995). Then from the slopes of *h*(*q, a*) and *D*(*q, a*) vs ln *a*, one gets *h*(*q*) and *D*(*q*) and therefore the *D*(*h*) singularity spectrum as a curve parametrized by *q*.

### 2.3. Monofractal vs. multifractal functions

Homogeneous *monofractal* functions, i.e., functions with singularities of unique Hölder exponent *H*, are characterized by a linear τ(*q*) curve of slope *H*. Monofractal scaling implies that the shape of the probability distribution function (pdf) of rescaled wavelet coefficients (*W*(·, *a*)/*a*^*H*^) does not depend on *a*, formally expressed by the self-similarity relationship (Arneodo et al., 2002):

(5) $$\rho_{W_{a}/a^{H}}(w)=\rho (w),$$

where *ρ*(*w*) is a “universal” pdf. A non-linear τ(*q*) is the signature of non-homogeneous *multifractal* functions, meaning that the Hölder exponent *h*(*t*) is a fluctuating quantity (Muzy et al., 1991, 1994; Arneodo et al., 1995) that depends on *t*. In this study, we fit the τ(*q*) data by the so-called log-normal quadratic approximation τ(*q*) = − *c*_0_ + *c*_1_*q* − *c*_2_*q*^2^/2, where the coefficients *c*_*n*_ > 0 (Delour et al., 2001). The corresponding singularity spectrum has a characteristic single-humped shape *D*(*h*) = *c*_0_ − (*h* − *c*_1_)^2^/2*c*_2_, where *c*_0_ = − τ(0) is the fractal dimension of the support of singularities of Σ, *c*_1_ is the value of *h* that maximizes *D*(*h*) and *c*_2_, the so-called *intermittency coefficient* (Muzy et al., 1991, 1994; Arneodo et al., 1995; Delour et al., 2001), characterizes the width of the *D*(*h*) spectrum, i.e., the signature of a change in WT coefficient statistics across scales (Muzy et al., 1991, 1994; Arneodo et al., 1995, 2002).

## 3. Description of data

### 3.1. Study design and population

Subjects were recruited for the present study from the Perm Regional Oncological Dispensary using procedures approved by the Local Ethics Committee (Gileva et al., 2012). They all gave Informed Consent to participate in this study via the recording of the IR thermograms of the both mammary glands, the cancerous one and the opposite undiagnosed one with no visible signs of pathology. Our database includes 33 females with ages between 37 and 83 (average 57 years) who all went through surgery to remove the histologically confirmed malignant tumor (invasive ductal and/or lobular breast cancer) a few weeks after thermograms were recorded. The tumors were found at different depths from 1 cm down to 12 cm with a size varying from 1.2 cm up to 6.5 cm (Table 1). As a control, we also investigated 14 women with intact mammary glands and of ages between 23 and 79 (average 49.6 years). This extensive study was preceded and encouraged by a preliminary study with only 6 patients and 3 volunteers (Gerasimova et al., 2013).

**Table 1:** **Set of (33) analyzed patients with age, cancerous breast (Right or Left), stage, size and depth of the malignant tumor and histology status**.

	**Age**	**Breast**	**Stage**	**Size (cm)**	**Depth (cm)**	**Location**	**Histological DS**
1	58	L	IIIa	1.57	4	Border outer-inner upper quadrants	Invasive ductal cancer
2	70	R	IIa	2.8	3	Border outer-inner upper quadrants	Invasive ductal cancer
3	53	R	IIa	2.5 × 2.85	5	Upper outer quadrant	Invasive ductal cancer
4	65	L	IIa	2.6 × 3.3	2	Border lower-upper outer quadrant	Cystadenocarcinoma
5	49	L	IIa	4.15 × 3.45	1	Border inner-outer upper quadrants	Cystadenocarcinoma
6	56	L	III	4.5	4	Upper outer quadrant	Invasive lobular cancer
7	47	R	IIa	2.7 × 2.2	3	Border inner-outer upper quadrants	Invasive lobular cancer
8	82	L	III	1.6 × 1.8	3	Upper inner quadrant	Invasive ductal cancer
9	44	L	IIa	1.8 × 3	6	Upper outer quadrant	Invasive ductal cancer
10	64	R	IIa	–	–		Invasive ductal cancer
11	55	R	IIb	5.3	1	Upper quadrant	Ductal cancer
12	59	R	Ib	1.8 and 1.26	12	Border between upper quadrants	Invasive ductal and lobular cancer
13	48	L	IIa	1.5	1	Underarm area	Invasive lobular cancer
14	73	R	I	1.2	5	Border between upper quadrants	Invasive ductal cancer
15	81	R	I	–	–		Invasive ductal cancer
16	41	R	IIa	3.4	7	Upper inner quadrant	Invasive lobular cancer
17	53	R	IIa	3 × 1.5 × 1.4	4	Upper outer quadrant	Invasive lobular and ductal cancer
18	37	L	IIa	3.49 × 2.39	6	Upper outer quadrant	Invasive ductal cancer
19	63	L	–	–	–		Invasive ductal cancer
20	56	R	IIa	3.1 × 2.5 × 3.6	3	Upper outer quadrant	Invasive lobular and ductal cancer
21	45	R	IIIb	6.5 × 4	2	Upper outer qadrant	Invasive cancer
22	74	R	IIa	–	–		Invasive ductal cancer
23	40	L	–	2.9 × 3.4	1	Border between upper quadrants	Invasive lobular cancer
24	49	L	IIa	1.7 × 2.2	2	Border between outer quadrants	Paget disease of the nipple
25	41	L	IIa	2.5 and 1.8 × 2, 2.7 × 1.7	3	Border between upper quadrants, upper outer quadrant	Invasive ductal cancer
26	83	R	Susp. ms	1.9	1	Underarm area	No metastasis
27	62	L	IIa	2.2 × 2.1	2	Border between inner quadrants	Invasive ductal and lobular cancer
28	76	L	IIb	3.49	8	Border between lower quadrants	Invasive ductal cancer
29	57	R	II	2.1 × 1.6	6	Border between lower quadrants	Invasive ductal cancer
30	54	R	IIa	3.5	3	Upper outer quadrant	Invasive ductal cancer
31	56	R	IIb	–	–		Invasive ductal cancer
32	55	R	IIb	4.4 × 3 × 2.5	9	Border between upper quadrants	Invasive ductal cancer
33	37	R	IIa	1.57	1.5	Border Upper-lower outer quadrants, close to the nipple	Invasive lobular cancer

### 3.2. IR thermography imaging

Both breasts of healthy volunteers and patients with breast cancer were imaged with a InSb photovoltaic (PV) detector camera (Joro et al., 2008a). Imaging was performed with the patient in sitting position with arms down to avoid too much discomfort during imaging. Frontal images were taken at a distance ~1 m of the patient in an environmental room temperature of 20–22°C. The image frame rate was set to 50 Hz. The image data were collected in 14 bits into the computer connected to the PV camera. Each image set comprised 30 000 256 × 320 pixel^2^ image frames during the 10 min immobile imaging phase. To eliminate low frequency patient movements, skin surface markers were successfully used as reference points for motion correction in the analysis.

### 3.3. Data sampling

**Figure 1:**
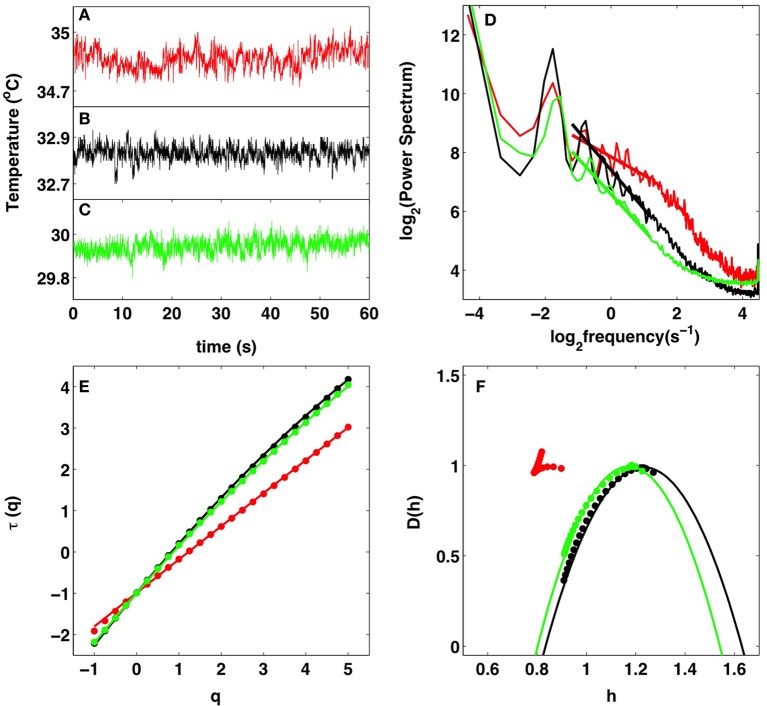
**Multifractal analysis of IR temperature time-series**. Comparative analysis of the cancerous right breast (red) and healthy left breast (black) of patient 20 (age 56) and of the healthy right breast (green) of volunteer 14 (age 60). **(A–C)** 1 min portions of pixel temperature time-series. **(D)** Averaged temperature power spectra in a 8 × 8 pixel^2^ square. The straight lines correspond to power-law scaling 1/*f*^β^ with exponent β = τ(2) = 0.62 (red), 1.32 (black), and 1.22 (green) as estimated with the WTMM method in **(E)**. **(E)** τ(*q*) vs. *q* estimated by linear regression fit of log_2_*Z*(*q, a*) vs. log_2_ *a* over a range of time-scales [0.3, 3] s (Figure 2A). **(F)** *D*(*h*) vs. *h* (Figures 2B,C). The solid lines in **(E,F)** correspond to quadratic spectra (see text) with parameters [*c*_0_, *c*_1_, *c*_2_] = [0.99, 0.81, 0.0044] (red), [0.99, 1.23, 0.080] (black), and [0.99, 1.171, 0.069] (green). In **(E,F)**, the τ(*q*) and *D*(*h*) spectra were averaged over a 8 × 8 pixel^2^ square.

Pixel based and windowed regional power spectra and wavelet-based multifractal analysis of normal and cancer breasts were tested to define the best procedure to minimize the effect of the camera noise and to ensure statistical convergence in the multifractal spectra estimation. We grouped single-pixel temperature time-series (Figures 1A–C) into 8 × 8 pixel^2^ squares spanning 10 × 10 mm^2^ and covering the entire breast (see Figures 4A–C). The results reported correspond to averaged power spectra, partition functions, singularity spectra and WT pdfs over 64 temperature time-series in these 8 × 8 subareas.

### 3.4. Remark

As commonly done for noise signals (Muzy et al., 1994; Audit et al., 2002) and previously experienced when applying the 1D WTMM method to rainfall time-series (Venugopal et al., 2006), the wavelet analysis was performed on the cumulative (or integral) Σ of the temperature time-series (Supplementary Figure 2) using the second-order compactly supported version *ψ*^(2)^_(3)_ of the Mexican hat (Roux et al., 1999) (Supplementary Figure 1). Hence the singularities with possible negative Hölder exponent −1 < *h* < 0, become singularities with 0 < *h*_*c*_ = *h* + 1 < 1 in the cumulative.

### 3.5. Software and documentation

The numerical procedure to perform the WTMM analysis of 1D signals can be downloaded at http://perso.ens-lyon.fr/benjamin.audit/LastWave

LastWave is an open source software written in C. We recommend interested users to read the LastWave C-Application Programming Interface documentation and to contact the corresponding author to be directed to the part of the code of most relevance to them.

## 4. Results

### 4.1. Spectral analysis reveals scale-invariance properties in skin temperature dynamics in both cancerous and healthy breasts

We analyzed individual 1-pixel temperature time-series taken from 8 × 8 pixels^2^-squares covering the patients entire breasts. As expected, these time-series generally fluctuate at a higher temperature when recorded in the tumor region of a malignant breast (Figure 1A) than in a symmetrically positioned square on the opposite breast (Figure 1B) as well as on a healthy breast (Figure 1C). When averaging the corresponding power-spectra over the 64 pixels of the considered squares, we observed for the two breasts of patient 20 (age 56) and the healthy breast of volunteer 14 (age 60), a rather convincing 1/*f*^β^ power-law scaling over a range of frequencies that extends from the characteristic human respiratory frequency (≳0.3 Hz) up to the cross-over frequency (≲4 Hz) toward (instrumental) white noise (Figure 1D). As confirmed by the WTMM method (Figure 1E), the exponent β = τ (*q* = 2) = 0.62 ± 0.19 found in the malignant breast is smaller than in the opposite breast of patient 20, β = 1.32 ± 0.11, and in the volunteer 14 healthy breast, β = 1.22 ± 0.11. This difference looks quite significant and very promising in a discriminatory perspective. Unfortunately, the histograms of β values obtained for all 8 × 8 pixel^2^ squares covering 33 cancerous breasts, the 32 opposite breasts (patient 6 had mastectomy of right breast) and the 28 volunteer healthy breasts are quite similar (Supplementary Figure 3) with mean values β = 1.09 ± 0.01 (cancer), 1.14 ± 0.01 (opposite) and 1.14 ± 0.01 (healthy). Indeed these histograms extend over a rather wide range of β values: 0.5 ≲ β ≲ 1.9.

### 4.2. WTMM analysis discriminates between monofractal (tumor area) and multifractal (healthy area) temperature temporal fluctuations

**Figure 2:**
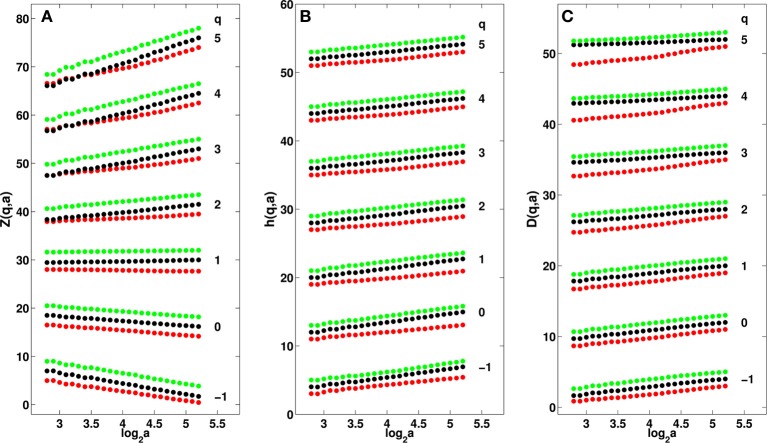
**Multifractal analysis of cumulative IR temperature time-series in a 8 × 8 pixel^2^ square in malignant right breast (red) and opposite left breast (black) of patient 20 and of the healthy right breast (green) of volunteer 14. (A)** log_2_*Z*(*q, a*) vs. log_2_(*a*) Equation (2). **(B)** *h*(*q, a*) vs. log_2_*a* Equation (4). **(C)** *D*(*q, a*) vs. log_2_ *a* Equation (4). The τ(*q*) spectra shown in Figure 1E were estimated by linear regression fit of the data in **(A)** over the range 2.8 ⩽ log_2_ *a* ⩽ 5.2. The *D*(*h*) spectra in Figure 1F were obtained by linear regression fit in **(B,C)** over the same range of time-scales. The analyzing wavelet is *ψ*^(2)^_(3)_ (Supplementary Figure 1).

**Figure 3:**
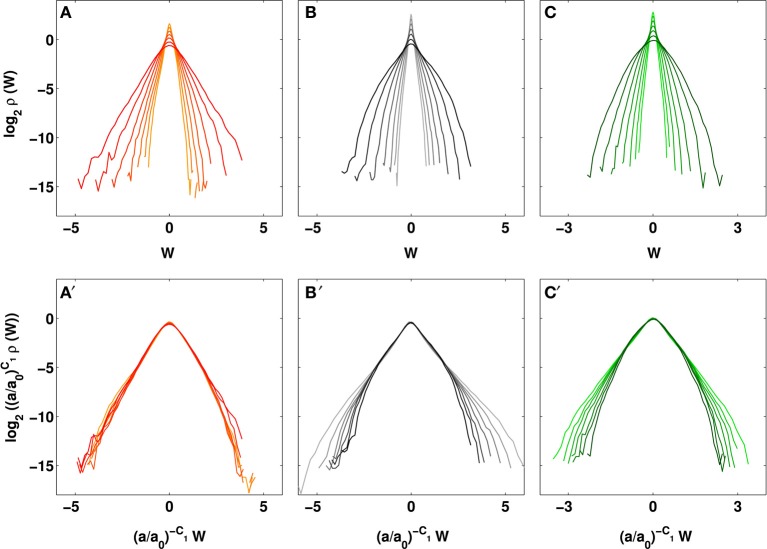
**Evolution of the temperature WT coefficient pdfs across time-scales**. Pdfs of WT coefficients *W* and rescaled WT coefficients (*W*/(*a*/*a*_0_)^*c*_1_^) of cumulative temperature of **(A,A′)** the cancerous right breast of patient 20: *c*_1_ = 0.81, **(B,B′)** the opposite left breast of patient 20: *c*_1_ = 1.23, and **(C,C′)** the healthy right breast of volunteer 14: *c*_1_ = 1.17. The different curves correspond to seven different scales from *a* = 0.43 s to 2.30 s, larger scales are in darker shades; *a*_0_ = 0.43 s. The pdfs were averaged over a 8 × 8 pixel^2^ square.

When applying the WTMM method to the cumulative of these temperature time-series, we confirmed that the partition functions *Z*(*q, a*) Equation (2) display nice scaling properties for *q* = − 1 to 5, over a range of time-scales that we strictly limited to (0.43, 2.30 s) for linear regression fit estimates in a logarithmic representation (Figure 2A). The τ(*q*) so-obtained are well approximated by quadratic spectra (Figure 1E). For the malignant breast of patient 20, τ(*q*) is nearly linear as quantified by a very small value of the intermittency coefficient *c*_2_ = (4.4 ± 0.6) · 10^−3^. This signature of monofractality is confirmed, when respectively plotting *h*(*q, a*) and *D*(*q, a*) Equation (4) vs. log_2_ *a*, in Figures 2B,C, where the slopes *h*(*q*) = *c*_1_ = 0.81 ± 0.01 and *D*(*q*) = 0.99 ± 0.03, do not significantly depend on *q*, meaning that the *D*(*h*) singularity spectrum nearly reduces to a single point *D*(*h* = *c*_1_ = 0.81) = 1 (Figure 1F). This monofractal diagnosis is confirmed when comparing the WT pdfs obtained at different time-scales (Figure 3A); according to Equation (5), they all collapse on a single curve when using the exponent *H* = *c*_1_ (Figure 3A′).

In contrast, the τ(*q*) spectrum obtained for the opposite breast of patient 20, is definitely non-linear with a no longer negligible (one order of magnitude larger) quadratic term *c*_2_ = 0.080 ± 0.001 (Figure 1E), the hallmark of multifractal scaling. As shown in Figures 2B,C, the slopes *h*(*q*) and *D*(*q*) of *h*(*q, a*) and *D*(*q, a*) vs log_2_ *a* now depend on *q*. From the estimate of *h*(*q*) and *D*(*q*), we get the single-humped *D*(*h*) spectrum shown in Figure 1F, which is well approximated by a quadratic spectrum with parameters *c*_0_ = 0.99 ± 0.05, *c*_1_ = 1.23 ± 0.01 and *c*_2_ = 0.080 ± 0.001. Because there is no longer a unique scaling exponent *c*_1_, the self-similarity Equation (5) is not verified meaning that the shape of the WT coefficient pdf now evolves across scales (Figure 3B), with fatter tails appearing at small scales (Figure 3B′). Interestingly, the τ(*q*) (Figure 1E) and *D*(*h*) (Figure 1F) spectra obtained for the healthy breast of volunteer 14 are quite similar quadratic spectra with parameter values *c*_0_ = 0.99 ± 0.03, *c*_1_ = 1.17 ± 0.01 and *c*_2_ = 0.069 ± 0.002. Again this multifractal diagnosis is strengthened by the observation that the WT coefficient pdf has a shape that evolves across time-scales (Figures 3C,C′).

To check the statistical relevance of our multifractal spectra estimates, we have generated so-called surrogate series (Theiler et al., 1992; Schreiber and Schmitz, 1996) that can be created with an identical pdf and optimally similar power spectrum to the original series (Supplementary Method). Examples of surrogate series for the IR temperature time-series shown in Figures 1A–C can be seen in Supplementary Figures 4A–C respectively. Visually, there is an obvious resemblance with the original time-series, but when computing the corresponding τ(*q*) (Supplementary Figure 4E) and *D*(*h*) (Supplementary Figure 4F), we find now for the three breasts a τ(*q*) spectrum that is quite linear and a *D*(*h*) singularity spectrum that almost reduces to a single point *D*(*h*) = *c*_1_ with a very small width *c*_2_ ≤ 0.01. This monofractal diagnostic is confirmed when reproducing this analysis for 100 surrogate series; the histograms of intermittency coefficients *c*_2_ obtained for the three breasts are very similar and mainly confined to very small values with means *c*_2_ = 0.012 (cancer), 0.005 (opposite) and 0.006 (healthy) that are all much smaller than the threshold *c*_2_ = 0.03 we will further use to discriminate between monofractal (*c*_2_ ≤ 0.03) and multifractal (*c*_2_ > 0.03) cumulative temperature time-series (Supplementary Figure 5). These results indicate that the cumulative temperature time-series of healthy breasts are not generated by an underlying linear Gaussian process, but have an inherently non-linear structure that is apparently lost in the presence of a malignant tumor.

### 4.3. Multifractal-based segmentation of breast thermograms into physiologically altered (risky) and normal regions

**Figure 4:**
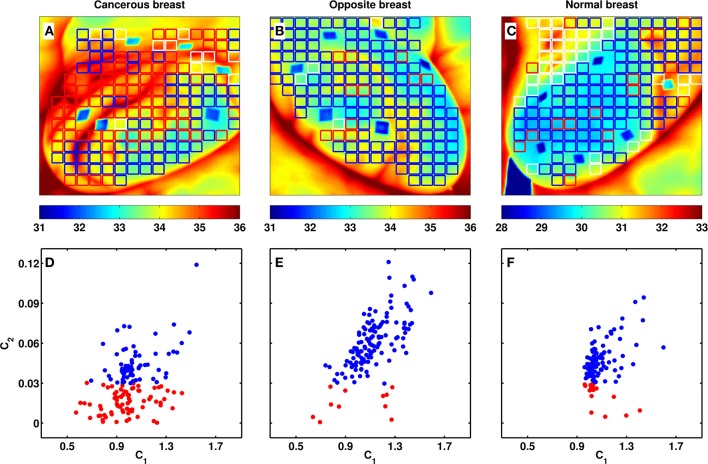
**Breast-wide multifractal analysis of skin temperature temporal fluctuations**. As estimated from the τ(*q*) spectra computed with the WTMM method (Figure 1E), 8 × 8 pixel^2^ squares covering the entire breast are colored coded **(A–C)** and represented as a dot in the (*c*_1_, *c*_2_) plane **(D–F)**. The colors have the following meaning: red: *c*_2_ < 0.03, blue: *c*_2_ ⩾ 0.03, and white: no scaling (see text). **(A,D)** Cancerous right breast of patient 20: the tumor is located in the upper outer quadrant (Table 1); **(B,E)** healthy left breast of patient 20; **(C,F)** healthy right breast of volunteer 14. The blue diamond shapes in **(A–C)** are skin surface markers used as references for motion correction.

When extending our wavelet-based multifractal analysis of cumulative temperature time-series to the entire set of 8 × 8 pixels^2^-squares that cover the right breast with malignant tumor of patient 20 (Figures 4A,D), her opposite left breast (Figures 4B,E) and the healthy right breast of volunteer 14 (Figures 4C,F), we confirmed, except in a minority of squares, the existence of scaling. In the cancerous breast, a large proportion of squares (49.7%) display monofractal temperature fluctuations with small intermittency coefficient values (*c*_2_ < 0.03 in Figure 4D), whereas only few of those squares are found in the opposite breast (7.7% in Figure 4E) and in the volunteer 14 healthy breast (11% in Figure 4F). Both these healthy breasts have a large majority of squares where multifractal scaling is observed (*c*_2_ ⩾ 0.03), namely 89.4% for the former (Figure 4B) and 65% for the latter (Figure 4C). Note that 43.1% of the squares in the cancerous breast also display multifractal temperature fluctuations as observed for healthy breasts (Figure 4A). These squares indeed cover regions of the breast that are far from the tumor area (left upper quadrant) mostly covered by monofractal squares. These results are indeed quite representative of the outcome of the statistical analysis of our entire data set.

### 4.4. Comparative statistical analysis of skin temperature dynamics in women breasts with and without malignant tumor

**Figure 5:**
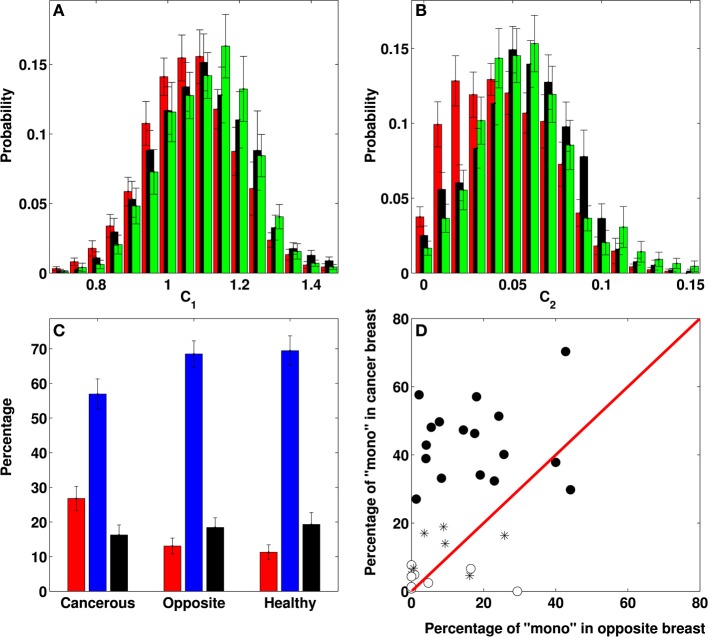
**Differential multifractal signature of temperature temporal fluctuations on breasts with and without tumor**. Comparative analysis of both breasts of 33 patients with breast cancer and 14 healthy volunteers. **(A)** Normalized histograms of *c*_1_ values obtained in the breasts with a malignant tumor (red: *N* = 4032 8 × 8 pixel^2^ squares), the opposite breasts (black: *N* = 3606) and the healthy breasts (green: *N* = 3185). **(B)** Normalized histograms of *c*_2_ values. **(C)** Percentage of “monofractal” (red: *c*_2_ < 0.03), “multifractal” (blue: *c*_2_ ⩾ 0.03) and “no scaling” (black) 8 × 8 pixel^2^ squares in cancerous, opposite and healthy breasts. **(D)** Percentage of “monofractal” 8 × 8 pixel^2^ squares in the cancerous breast vs. the percentage in the opposite breast of each patient; the symbols have the following meaning: (•) 17 patients with a percentage of monofractal squares on the cancerous breast larger than the mean value 26.8%, namely patients 2, 4, 7, 8, 9, 10, 11, 13, 17, 19, 20, 22, 25, 26, 29, 30, and 32 (patient 6 is not taken into account because of mastectomy of right breast); (*) 7 patients with the percentage of monofractal squares on the cancerous breast smaller than 26.8% but well localized on the tumor region, namely patients 3 (16.3%), 14 (18.9%), 21 (14.0%), 23 (4.6%), 24 (4.9%), 31 (6.8%), and 33 (17.0%); (°) 8 false negatives, namely patients 1 (2.4%), 5 (0.8%), 12 (4.9%), 15 (1.3%), 16 (0.0%), 18 (7.7%), 27 (4.3%), and 28 (6.6%) (see Table 2).

The results of our comparative wavelet-based multifractal analysis of (cumulative) temperature fluctuations over the 33 cancerous breasts, the 32 opposite breasts (no right breast for patient 6) and the 28 volunteer healthy breasts are reported in Figure 5 and Table 2. In Figure 5A, the corresponding histograms of *c*_1_ values extend over a rather wide range 0.6 ≲ *c*_1_ ≲ 1.8 but turn out to be quite similar with mean values *c*_1_ = 1.066 ± 0.002 (cancer), 1.104 ± 0.002 (opposite) and 1.103 ± 0.002 (healthy). This is reminiscent to the similar histograms that were also obtained for the power-spectrum exponent β (Supplementary Figure 3). In contrast, the intermittency parameter *c*_2_ has a definite discriminatory power. The histogram for cancerous breasts (*c*_2_ = 0.045 ± 0.001) is definitely shifted toward smaller values relative to the ones for opposite (*c*_2_ = 0.056 ± 0.001) and healthy breasts (*c*_2_ = 0.058 ± 0.001) (Figure 5B). The small-value left-side of the *c*_2_ histogram is much more populated in cancerous than in healthy breasts, confirming that cancerous breasts are enriched in squares where temperature fluctuations display significantly reduced multifractal properties. This justifies that we considered *c*_2_ = 0.03 as the threshold below (resp. above) which a square was qualified as monofractal (resp. multifractal) (Figure 4). Note that for each breast, a small percentage (≲20%) of (white) squares were removed from our analysis because of lack of scaling (Figure 5C, Table 2 and Supplementary Table 1). Importantly, the percentage of monofractal (red) squares covering cancerous breasts (26.8 ± 3.5%) is about twice larger than the ones covering opposite (13.1 ± 2.3%) and healthy (11.3 ± 2.2%) breasts. This excess is compensated by a smaller percentage of multifractal (blue) squares in cancerous breasts (56.9 ± 4.4%) than in opposite (68.5 ± 3.8%) and healthy (69.4 ± 4.3%) breasts (Figure 5C).

**Table 2:** **Results of our WTMM multifractal analysis of the skin (cumulative) temperature temporal fluctuations of the two breasts of our 33 patients with breast cancer**.

	**Cancerous breast**				**Opposite breast**			
	***N*_mono_**	***N*_multi_**	***N*_no scaling_**	**Total**	***N*_mono_**	***N*_multi_**	***N*_no scaling_**	**Total**
1	2 (2.4%)	20 (24.4%)	60 (73.2%)	82 (100%)	3 (4.7%)	46 (71.9%)	15 (23.4%)	64 (100%)
2	39 (42.9%)	23 (25.3%)	29 (31.9%)	91 (100%)	5 (4.1%)	61 (50.0%)	56 (45.9%)	122 (100%)
3	16 (16.3%)	60 (61.2%)	22 (22.4%)	98 (100%)	31 (25.8%)	77 (64.2%)	12 (10.0%)	120 (100%)
4	56 (33.1%)	90 (53.3%)	23 (13.6%)	169 (100%)	7 (8.3%)	62 (73.8%)	15 (17.9%)	84 (100%)
5	1 (0.8%)	108 (82.4%)	22 (16.8%)	131 (100%)	0 (0.0%)	122 (91.7%)	11 (8.3%)	133 (100%)
6	54 (30.5%)	101 (57.1%)	22 (12.4%)	177 (100%)				
7	44 (32.4%)	59 (43.4%)	33 (24.3%)	136 (100%)	26 (23.0%)	75 (66.4%)	12 (10.6%)	113 (100%)
8	20 (27.0%)	46 (62.2%)	8 (10.8%)	74 (100%)	1 (1.4%)	29 (39.2%)	44 (59.5%)	74 (100%)
9	65 (40.1%)	85 (52.5%)	12 (7.4%)	162 (100%)	39 (25.7%)	37 (24.3%)	76 (50.0%)	152 (100%)
10	77 (51.3%)	42 (28.0%)	31 (20.7%)	150 (100%)	40 (24.2%)	96 (58.2%)	29 (17.6%)	165 (100%)
11	62 (46.3%)	54 (40.3%)	18 (13.4%)	134 (100%)	23 (17.6%)	87 (66.4%)	21 (16.0%)	131 (100%)
12	5 (4.9%)	97 (94.2%)	1 (1.0%)	103 (100%)	1 (0.9%)	114 (99.1%)	0 (0.0%)	115 (100%)
13	48 (37.8%)	18 (14.2%)	61 (48.0%)	127 (100%)	50 (40.0%)	18 (14.4%)	57 (45.6%)	125 (100%)
14	17 (18.9%)	65 (72.2%)	8 (8.9%)	90 (100%)	8 (8.9%)	72 (80.0%)	10 (11.1%)	90 (100%)
15	1 (1.3%)	70 (90.9%)	6 (7.8%)	77 (100%)	0 (0.0%)	26 (89.7%)	3 (10.3%)	29 (100%)
16	0 (0.0%)	79 (54.1%)	67 (45.9%)	146 (100%)	52 (29.4%)	111 (62.7%)	14 (7.9%)	177 (100%)
17	106 (57.6%)	64 (34.8%)	14 (7.6%)	184 (100%)	3 (2.1%)	104 (73.8%)	34 (24.1%)	141 (100%)
18	11 (7.7%)	111 (77.6%)	21 (14.7%)	143 (100%)	0 (0.0%)	111 (93.3%)	8 (6.7%)	119 (100%)
19	60 (34.1%)	106 (60.2%)	10 (5.7%)	176 (100%)	29 (19.1%)	113 (74.3%)	10 (6.6%)	152 (100%)
20	76 (49.7%)	66 (43.1%)	11 (7.2%)	153 (100%)	11 (7.7%)	127 (89.4%)	4 (2.8%)	142 (100%)
21	25 (14.0%)	148 (82.7%)	6 (3.4%)	179 (100%)	17 (9.3%)	156 (85.7%)	9 (4.9%)	182 (100%)
22	50 (29.8%)	41 (24.4%)	77 (45.8%)	168 (100%)	86 (44.1%)	82 (42.1%)	27 (13.8%)	195 (100%)
23	7 (4.6%)	143 (93.5%)	3 (2.0%)	153 (100%)	27 (16.2%)	111 (66.5%)	29 (17.4%)	167 (100%)
24	8 (4.9%)	144 (88.3%)	11 (6.7%)	163 (100%)	0 (0.0%)	163 (94.2%)	10 (5.8%)	173 (100%)
25	77 (57.0%)	55 (40.7%)	3 (2.2%)	135 (100%)	24 (18.0%)	101 (75.9%)	8 (6.0%)	133 (100%)
26	87 (47.3%)	31 (16.8%)	66 (35.9%)	184 (100%)	18 (14.4%)	37 (29.6%)	70 (56.0%)	125 (100%)
27	7 (4.3%)	148 (91.4%)	7 (4.3%)	162 (100%)	0 (0.0%)	139 (91.4%)	13 (8.6%)	152 (100%)
28	11 (6.6%)	140 (84.3%)	15 (9.0%)	166 (100%)	27 (16.5%)	84 (51.2%)	53 (32.3%)	164 (100%)
29	65 (38.9%)	67 (40.1%)	35 (21.0%)	167 (100%)	7 (4.0%)	144 (82.8%)	23 (13.2%)	174 (100%)
30	116 (70.3%)	40 (24.2%)	9 (5.5%)	165 (100%)	65 (42.8%)	72 (47.4%)	15 (9.9%)	152 (100%)
31	13 (6.8%)	175 (91.1%)	4 (2.1%)	192 (100%)	1 (0.6%)	140 (82.8%)	28 (16.6%)	169 (100%)
32	88 (48.1%)	90 (49.2%)	5 (2.7%)	183 (100%)	11 (5.5%)	160 (80.0%)	29 (14.5%)	200 (100%)
33	23 (17.0%)	109 (80.7%)	3 (2.2%)	135 (100%)	5 (3.6%)	112 (80.0%)	23 (16.4%)	140 (100%)

*Number and percentage of monofractal (*c*_2_ < 0.03), multifractal (*c*_2_ ⩾ 0.03), no-scaling (and total) 8 × 8 pixel^2^ squares in the cancerous breast and in the opposite breast (see Figure 4 and Supplementary Figures 4–7)*.

A common way to suspect cancer by IR thermography is to look for some dissymmetry between the two breasts of a patient (Etehadtavakol and Ng, 2013). When comparing the percentages of monofractal squares on both the cancer and opposite breasts of the 33 patients (except patient 6), we found that 25 (/32) have more monofractal squares on the cancerous breast (Table 2). Indeed, we found 18 (/33) malignant breasts that have a percentage of monofractal squares greater than the mean value 26.8 ± 3.5 (Figure 5D). Among the other 15 cancerous breasts, 7 have a smaller percentage of monofractal squares but well localized on the tumor region (Figure 4A and Supplementary Figure 6). The remaining 8 cancerous breasts correspond to false negatives for which not only the percentage of monofractal squares is small but their location is far from the tumor region (Supplementary Figure 7). Among these false negatives, 4 correspond to rather deep tumors in fatty breasts which can explain that they do not manifest in a qualitative change in temperature dynamics at the skin surface in patients 12 (size 1.8 cm, depth 12 cm) (Supplementary Figure 8), 16 (3.4 cm, 7 cm), 18 (3.49 cm, 6 cm) and 28 (3.49 cm, 8 cm). When investigating the 32 opposite breasts, 5 of them have a large percentage of monofractal squares (Figure 5D, Table 2 and Supplementary Figure 9). These important percentages similar to those obtained in malignant breasts are probable indication of some physiological changes in the opposite breast that may announce the possible extension of cancer to the second breast. As a control, we reproduced this comparative analysis on the two breasts of the 14 healthy volunteers (Supplementary Figure 10 and Table 1). Among the 28 breasts analyzed, only 4 have a large (⩾26.8%) percentage of monofractal squares, whereas most of them have a percentage ≲10% (Supplementary Figures 11, 12). Overall, we thus obtained 25 (/33) true positives and 4 (/28) false positives, i.e., a sensitivity of 76% and a specificity of 86%, respectively.

## 5. Conclusions

Over the course of a lifetime, 1 in 8 women will be diagnosed with breast cancer. There are no well-established ways to avoid breast cancer (as opposed to lung cancer for example) and in the context of breast cancer screening, abnormalities should be detected at an early stage to improve prognosis. Criticism of the use of screening mammography due to over-diagnosis led some researchers to show that one in three breast cancers identified by mammography would not cause symptoms in a patient's lifetime (Jorgensen and Gotzsche, 2009). Therefore, alternative and accurate screening technologies must be developed. The functional and technical background of dynamic IR imaging has the potential for early detection of breast cancer and treatment response evaluation if optimal diagnostic algorithms are developed. We have shown that the wavelet-based multifractal analysis of dynamic IR thermograms is able to discriminate between cancerous breasts with monofractal (cumulative) temperature temporal fluctuations characterized by a unique singularity exponent (*h* = *c*_1_), and healthy breasts with multifractal temperature fluctuations requiring a wide range of singularity exponents as quantified by the intermittency coefficient *c*_2_ ≫ 0. This is strikingly analogous to the results of a similar wavelet-based analysis of human heart beat dynamics (Ivanov et al., 1999, 2001; Goldberger et al., 2002), where the multifractal character and non-linear properties of the healthy heart rate were shown to be lost in pathological condition, congestive heart failure. Indeed, this distinction was intrinsically beyond the capability of spectral (Fourier) analysis which only gives access to the power-spectrum exponent β = τ(*q* = 2) = −*c*_0_ + 2*c*_1_ − 2*c*_2_, and not to the full τ(*q*) spectrum required for multifractal diagnosis (Muzy et al., 1991, 1994; Arneodo et al., 1995, 2008). Furthermore the fact that *c*_1_ (~ 1) is about one order of magnitude larger than the intermittency coefficient *c*_2_ (≲ 0.1), explains why very much like *c*_1_ (Figure 5A), the spectral exponent β ~ −*c*_0_ + 2*c*_1_ ~ 1 ≫ *c*_2_ (Supplementary Figure 3) has no discriminatory power.

Interdisciplinary effort revealing specific fractal characteristics for healthy and cancerous breast tissues definitely challenges current knowledge in physical, physiological and clinical fundamentals of oncogenesis. Fundamentally, our results indicate that skin temperature fluctuations of healthy breasts are more complex (multifractal) than previously suspected. They definitely raise new challenging questions to ongoing efforts to develop physiological 3D breast models that account for the skin surface temperature distribution in the presence (or absence) of an internal tumor (Ng and Sudharsan, 2004; Xu et al., 2008; Lin et al., 2009). The observed drastic simplification from multifractal to monofractal skin temperature dynamics may result from some increase in blood flow and cellular activity associated with the presence of a tumor (Thomsen and Miles, 1998; Anbar et al., 2001; Button et al., 2004; Joro et al., 2008b). More likely it can be the signature of some architectural change in the cellular microenvironment of the breast tumor (Bissell and Hines, 2011) that may deeply affect heat transfer and related thermomechanics in breast tissue (Xu et al., 2008; Quail and Joyce, 2013). Identifying the regulation mechanisms that originate in a loss of multifractal temperature dynamics will be an important step toward understanding breast cancer development, tumor growth and progression. Dynamic IR thermography is a non-invasive and objective screening method that is inexpensive, quick and painless for the patient. Future use of wavelet-based multifractal processing of dynamic IR thermography, could help identifying women with high risk of breast cancer, prior to more traumatic and painful examination such as mammography and biopsy. It can also prove to be a valuable and reliable adjunct tool for early detection of tumors in other locations than in mammary glands.

## Grant support

This work was supported by INSERM, ITMO Cancer for its financial support under contract PC201201-084862 “Physiques, mathématiques ou sciences de l'ingénieur appliqués au Cancer,” the Perm Regional Government (Russia) for the contract “Multiscale approaches in mechanobiology for early cancer diagnosis” and the Maine Cancer Foundation.

### Conflict of interest statement

The authors declare that the research was conducted in the absence of any commercial or financial relationships that could be construed as a potential conflict of interest.
