# Supplementary material for: Wavelet-based multifractal analysis of dynamic infrared thermograms to assist in early breast cancer diagnosis
Source: Front Physiol. 2014 May 8;5:176. doi: 10.3389/fphys.2014.00176 (PMC4021111; doi:10.3389/fphys.2014.00176)
Supplement: Supplementary file 1 [file Presentation1.PDF]

Frontiers in Physiology

Original Research Article
10 April 2014

Supplementary Material: Wavelet-based multifractal analysis of dynamic infrared thermograms to assist in early breast cancer diagnosis

Evgeniya Gerasimova [1], Benjamin Audit [2], Stephane-G. Roux [2], André Khalil [3], Olga Gileva [4], Françoise Argoul [2], Oleg Naimark [1] and Alain Arneodo [2,*]

[1] *Laboratory of Physical Foundation of Strength, Institute of Continuous Media Mechanics UB RAS, Perm, Russia*
[2] *Université de Lyon and Laboratoire de Physique, ENS de Lyon, CNRS, UMR 5672, Lyon, France*
[3] *University of Maine, Department of Mathematics and Statistics, Orono, Maine, USA*
[4] *Perm State Academy of Medicine, Department of Therapeutic and Propedeutic Dentistry, Perm, Russia*

Correspondence*:
Alain Arneodo
Laboratoire de Physique, CNRS, ENS de Lyon, 46 Alleé d'Italie, Lyon, F-69007, France, alain.arneodo@ens-lyon.fr

1 SUPPLEMENTARY METHOD

SURROGATES

A classical method to test the statistical significance of the multifractal spectra estimation is to generate to the so-called surrogate series (1, 2). Hypothesis testing is typically performed by evaluating some test statistic, or measure of nonlinearity, for both the original series and an ensemble of surrogate series. The results for the ensemble of surrogates provide the distribution of the test statistic that would be produced by an inherently linear process. This allows the establishment of confidence intervals for the rejection of the null hypothesis based on the value of the test statistic computed from the original series.

The surrogate series $\{s_n\}$ is assumed to be generated by a process of the form

$$s_n = S(T_n) \ , \quad T_n = \sum_{i=1}^{M} a_i T_{n-i} + \sum_{i=0}^{N} b_i \eta_{n-i}$$

where S could be any invertible nonlinear function. $\{T_n\}$ is the underlying linear process, $\{a_n\}$ and $\{b_n\}$ are constant coefficients and $\{\eta_n\}$ is white Gaussian noise. M and N are the orders of an autoregressive (first term) and moving average (second term) model, respectively. To generate surrogates that maintain the pdf and correlation structure (and hence power spectrum) of the original data, we use the method proposed by Schreiber and Schmitz (2), known as the iterative amplitude adjured Fourier transform (IAAFT) method. This is a modification of the earlier amplitude adjusted Fourier transform (AAFT) method, that iteratively adjusts both pdf and linear correlation structure to minimize their deviation from the original series. The generation process proceeds in the following way:

1. Randomly shuffle the data points of the original series $\{T_n\}$ to destroy any correlation or nonlinear relationships, while keeping the pdf unchanged. The reshuffled series is the starting point for the iteration $\{s_n^{(0)}\}$.
2. Take the Fourier transform of the current series $\{T_n^{(i)}\}$, and adjust the amplitudes to recreate the power spectrum of the original data. Keep the phases unchanged. Perform inverse Fourier transform.
3. The pdf will no longer be correct. Transform the data to the correct pdf by rank ordering and replacing each value with the value in the original series $\{T_n\}$ with the same rank. This gives the updated series $\{s_n^{(i+1)}\}$.
4. Repeat steps 2 and 3 until the discrepancy in the power spectrum is below a threshold, or the sequence stops changing (reaches a fixed point).

In this manner a surrogate data series can be created with an identical pdf and optimally similar power spectrum to the original series. Any underlying nonlinear structure, which in Fourier space would be embodied by correlations in the phase, is destroyed, since only the absolute value (or power) of the Fourier coefficients is retained, whereas the phases are randomized by the shuffling of the series. For a heuristic argument for the convergence of the algorithm, see Schreiber and Schmitz (2).

REFERENCES

1 .Theiler J, Eubank S, Longtin A, Galdikian B, Farmer JD. Testing for nonlinearity in time series: the method of surrogate data. *Physica D* **58** (1992) 77-94.

2 .Schreiber T, Schmitz A. Improved surrogate data for nonlinear tests. *Phys. Rev. Lett.* **77** (1996) 635-638.

2 SUPPLEMENTARY TABLES AND FIGURES

Supplementary Table 1. Results of our WTMM multifractal analysis of the skin (cumulative) temperature temporal fluctuations of the two breasts of our 14 healthy volunteers. Number and percentage of monofractal ($c_2 < 0.03$), multifractal ($c_2 \geqslant 0.03$), no-scaling (and total) 8×8 pixel^2 squares in the right and left breasts (see Fig. 4 and Supplementary Figs 9, 10).

		Right breast				Left breast			
	**Age**	$\mathbf{N}_{mono}$	$\mathbf{N}_{multi}$	$\mathbf{N}_{noscaling}$	**Total**	$\mathbf{N}_{mono}$	$\mathbf{N}_{multi}$	$\mathbf{N}_{noscaling}$	**Total**
**1**	44	10 (5.8%)	157 (91.3%)	5 (2.9%)	172 (100 %)	7 (4.0%)	163 (92.1%)	7 (4.0%)	177 (100 %)
**2**	64	8 (5.7%)	100 (71.4%)	32 (22.9%)	140 (100 %)	13 (9.0%)	93 (64.1%)	39 (26.9%)	145 (100 %)
**3**	26	0 (0.0%)	99 (97.1%)	3 (2.9%)	102 (100 %)	24 (20.9%)	76 (66.1%)	15 (13.0%)	115 (100 %)
**4**	53	38 (42.7%)	24 (27.0%)	27 (30.3%)	89 (100 %)	12 (13.2%)	50 (54.9%)	29 (31.9%)	91 (100 %)
**5**	26	7 (3.8%)	168 (90.8%)	10 (5.4%)	185 (100 %)	3 (1.7%)	148 (85.5%)	22 (12.7%)	173 (100 %)
**6**	35	30 (25.2%)	55 (46.2%)	34 (28.6%)	119 (100 %)	29 (20.7%)	82 (58.6%)	29 (20.7%)	140 (100 %)
**7**	61	11 (7.4%)	117 (79.1%)	20 (13.5%)	148 (100 %)	36 (20.5%)	127 (72.2%)	13 (7.4%)	176 (100 %)
**8**	66	12 (10.2%)	91 (77.1%)	15 (12.7%)	118 (100 %)	41 (31.8%)	42 (32.6%)	46 (35.7%)	129 (100 %)
**9**	79	0 (0.0%)	3 (2.7%)	110 (97.3%)	113 (100 %)	0 (0.0%)	81 (87.1%)	12 (12.9%)	93 (100 %)
**10**	55	3 (2.2%)	126 (92.6%)	7 (5.1%)	136 (100 %)	2 (1.9%)	96 (91.4%)	7 (6.7%)	105 (100 %)
**11**	55	5 (3.3%)	137 (90.1%)	10 (6.6%)	152 (100 %)	15 (9.7%)	125 (81.2%)	14 (9.1%)	154 (100 %)
**12**	47	3 (2.0%)	99 (66.4%)	47 (31.5%)	149 (100 %)	2 (1.4%)	111 (76.0%)	33 (22.6%)	146 (100 %)
**13**	23	49 (37.1%)	50 (37.9%)	33 (25.0%)	132 (100 %)	28 (16.1%)	105 (60.3%)	41 (23.6%)	174 (100 %)
**14**	60	16 (10.7%)	98 (65.3%)	36 (24.0%)	150 (100 %)	14 (8.5%)	144 (87.3%)	7 (4.2%)	165 (100 %)

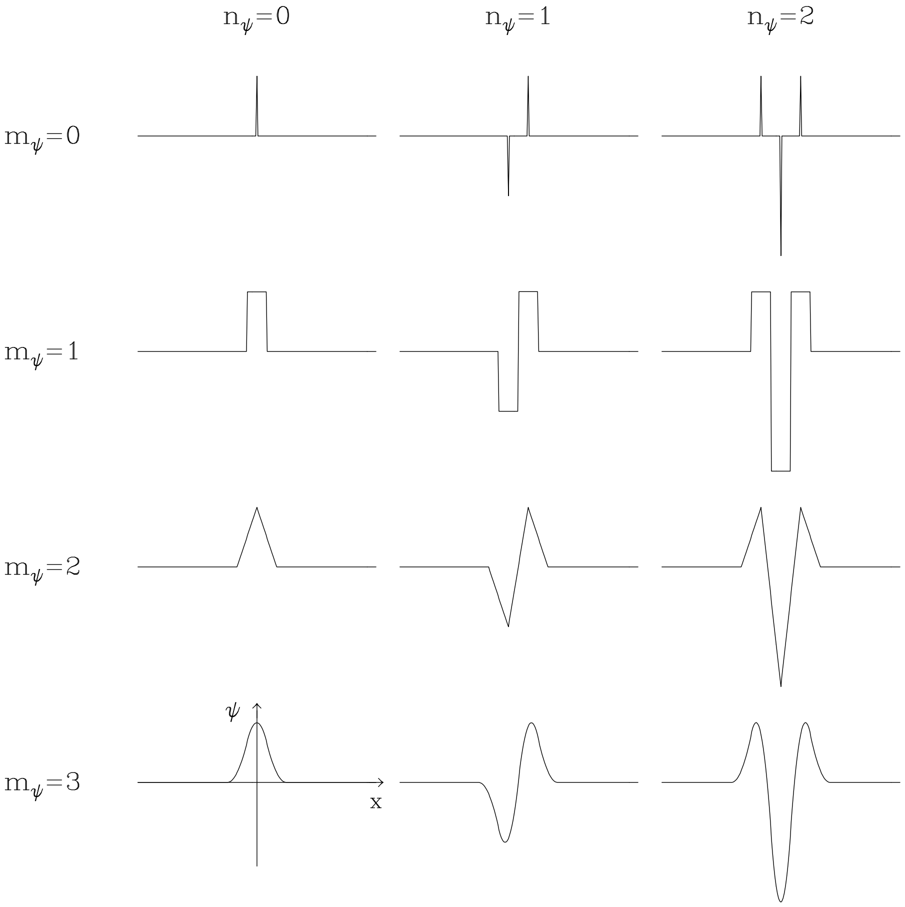

Supplementary Figure 1. Set of compactly supported analyzing wavelets $\psi_{(m)}^{(n)}$. n_ψ correspond to the number of vanishing moments. The function $\psi_{(m)}^{(n)}$ are smooth versions of $\psi_{(0)}^{(n)}$ obtained after m successive convolutions with the box function χ.

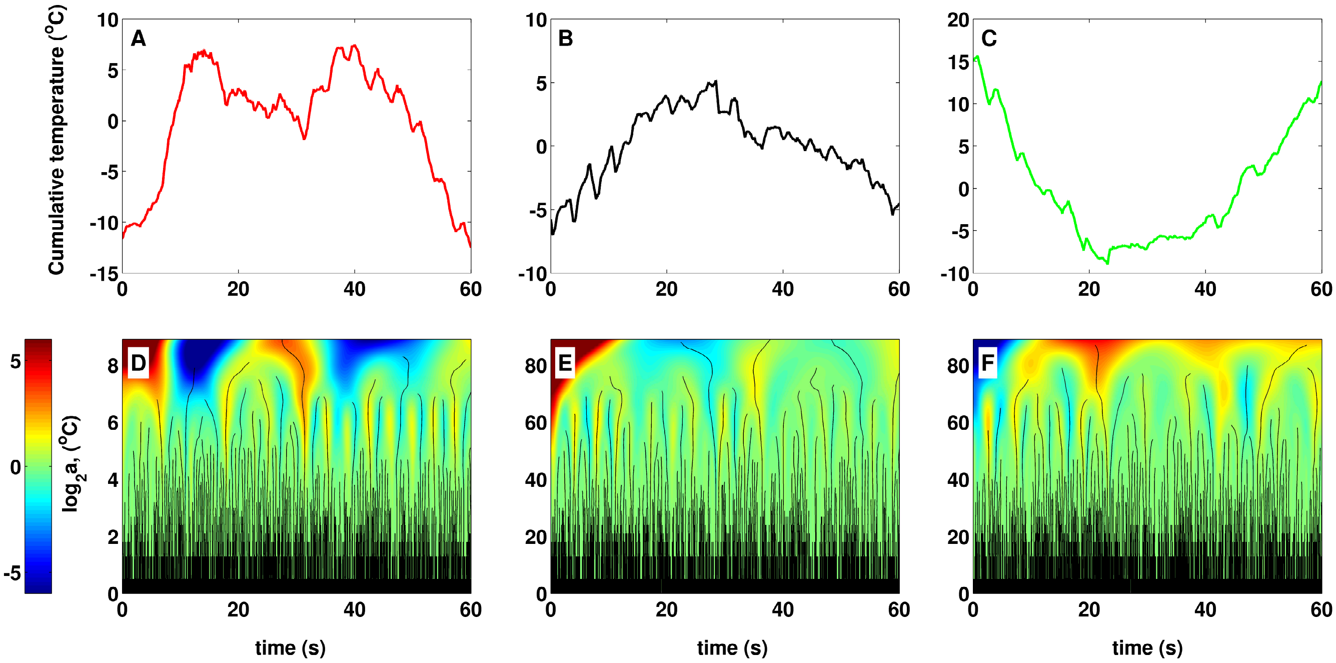

Supplementary Figure 2. Comparative wavelet analysis of the cancerous right breast (red) and the opposite left breast (black) of patient 20 (age 56) and of the healthy right breast (green) of volunteer 14 (age 60). (**A–C**) 1 min portion of cumulative pixel temperature time-series (after removing the overall linear trend). (**D–F**) WT of the cumulative time-series as coded, from black (min $|W(\cdot, a)|$) to red (max $W(\cdot, a)$); solid black lines are the WTMM lines that define the WT skeleton. The analyzing wavelet is the second-order compactly supported analyzing wavelet $\psi_{(3)}^{(2)}$ (Fig. 1).

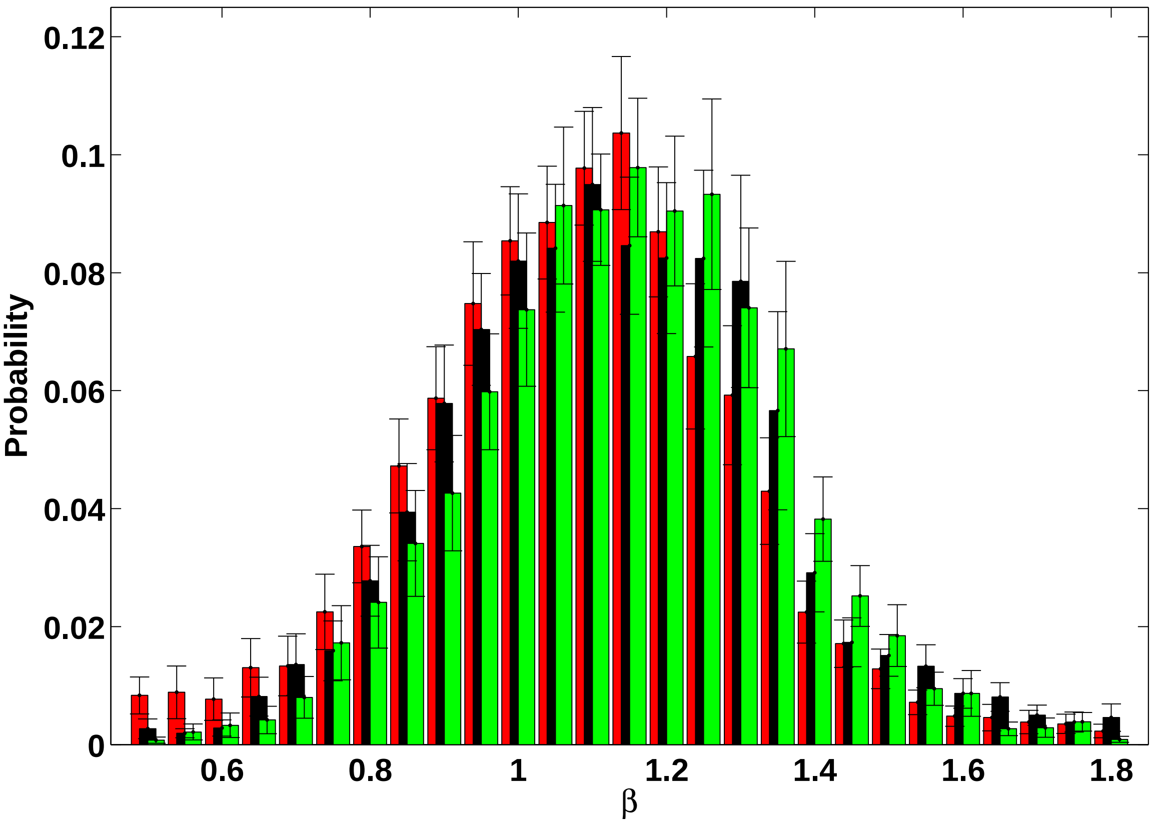

Supplementary Figure 3. Comparative power-spectrum analysis of temperature time-series recorded on both breasts of 33 patients with breast cancer and 14 healthy volunteers. Normalized histograms of power-law scaling exponent $\beta = \tau(2)$ values obtained in the breast with malignant tumor (red: $N = 4032$ 8×8 pixel^2 squares), the opposite breasts (black: $N = 3606$) and the healthy breasts (green: $N = 3185$).

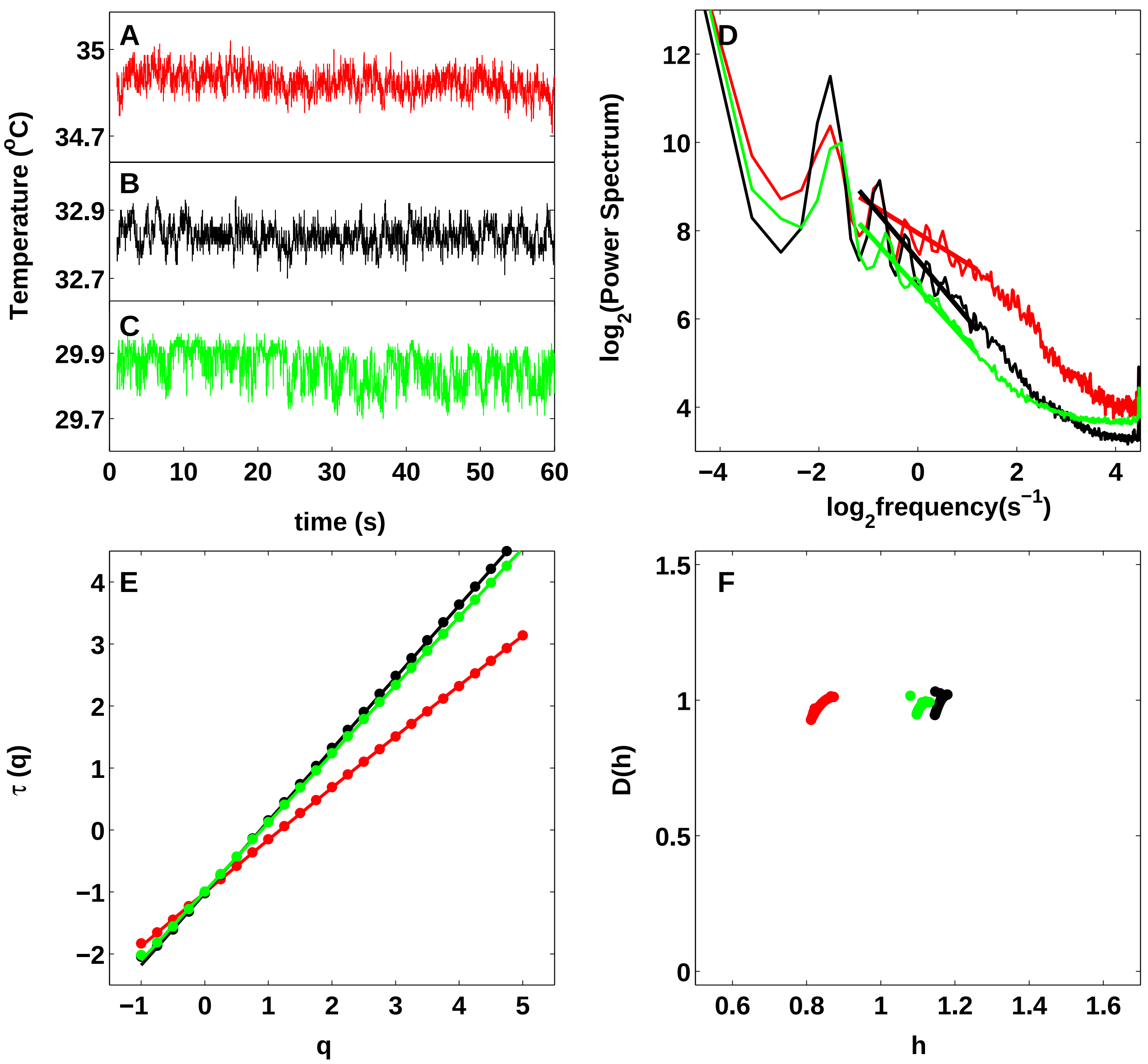

Supplementary Figure 4. Multifractal analysis of one surrogate realization of the IR temperature time-series shown in Fig. 1A, B, C. Comparative analysis of the cancerous right breast (red) and healthy left breast (black) of patient 20 (age 56) and of the healthy right breast (green) of volunteer 14 (age 60). (A–C) 1 min portions of pixel surrogate temperature time-series. (D) Averaged temperature power spectra in a 8×8 pixel^2 square. The straight lines correspond to power-law scaling $1/f^{\beta}$ with exponent $\beta = \tau(2) = 0.69$ (red), 1.33 (black) and 1.24 (green) as estimated with the WTMM method in (E). (E) $\tau(q)$ vs q estimated by linear regression fit of $\log_2 Z(q, a)$ vs $\log_2 a$ over a range of time-scales [0.3, 3] s. (F) $D(h)$ vs h. The solid lines in (E) correspond to quadratic spectra (see text) with parameters $[c_0, c_1, c_2] = [1.01, 0.86, 0.011]$ (red), $[1.02, 1.16, 0.001]$ (black) and $[0.99, 1.11, 0.001]$ (green). In (E) and (F), the $\tau(q)$ and $D(h)$ spectra were averaged over a 8×8 pixel^2 square.

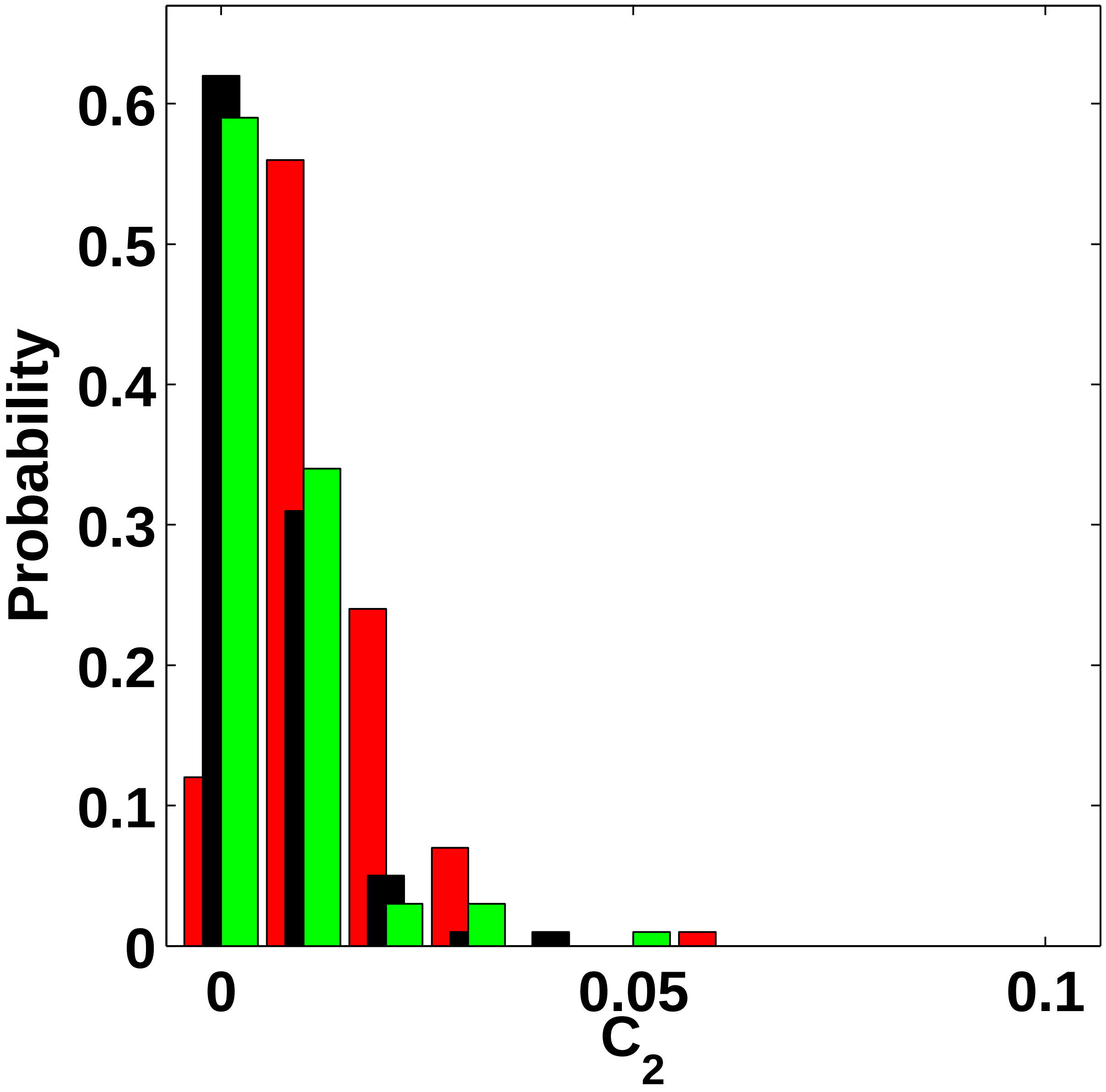

Supplementary Figure 5. Normalized histogram of c_2 values obtained from the multifractal analysis of 100 surrogate realizations of the 8×8 pixel^2 square IR temperature time-series from the cancerous right breast (red) and healthy left breast (black) of patient 20 (age 56) and of the healthy right breast (green) of volunteer 14 (age 60).

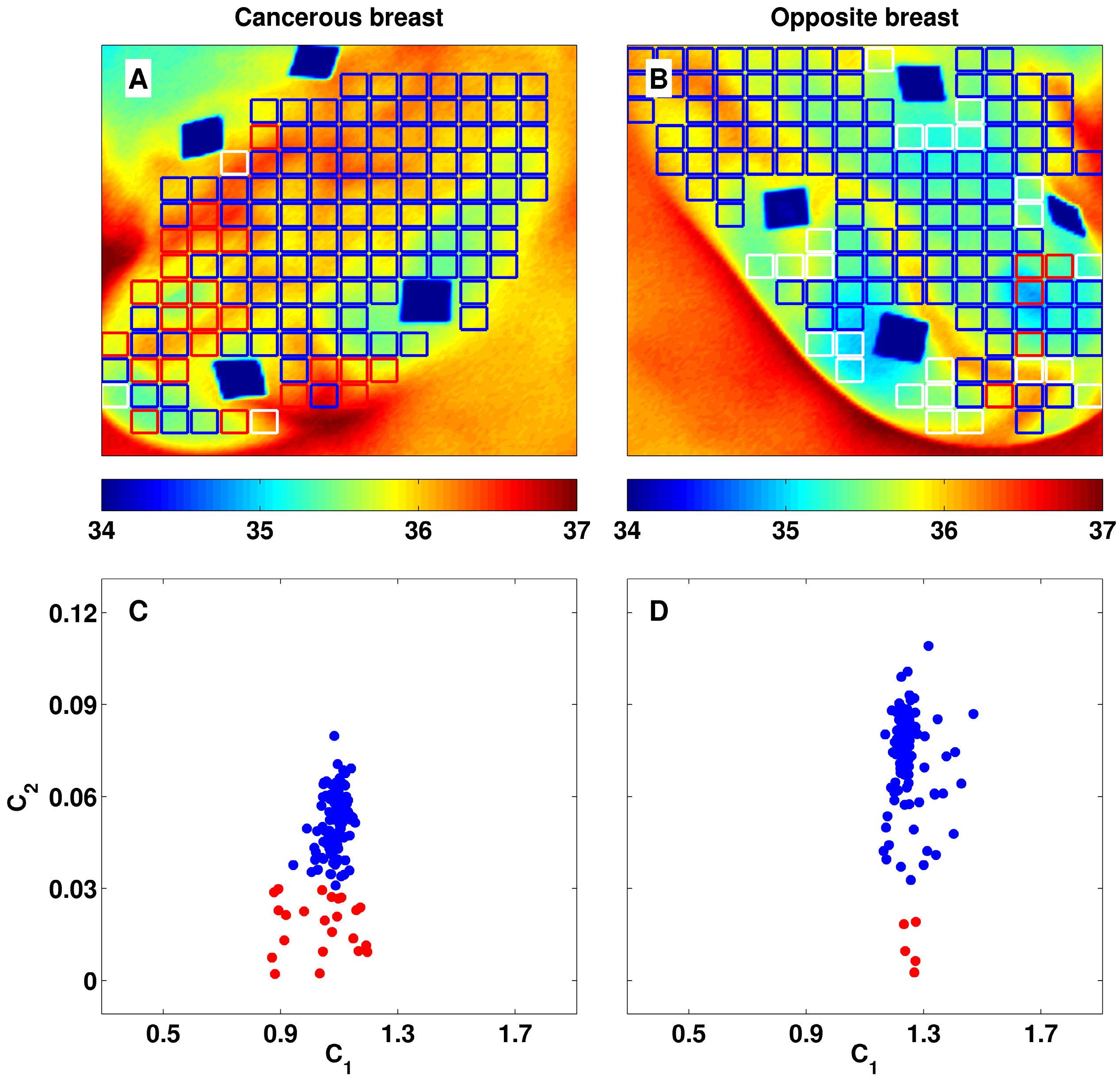

Supplementary Figure 6. Breast-wide multifractal analysis of skin (cumulative) temperature temporal fluctuations. As estimated from the $\tau(q)$ spectra computed with the WTMM method (Figs 1 and 2), 8×8 pixel^2 squares covering the two breasts of patient 33 (age 37) are color coded (A, B) and represented as a dot in the (c_1, c_2) plane (C, D). The colors have the following meaning: $c_2 < 0.03$ (red), $c_2 \geqslant 0.03$ (blue) and no scaling (white). (A, C) Cancerous right breast; (B, D) opposite left breast.

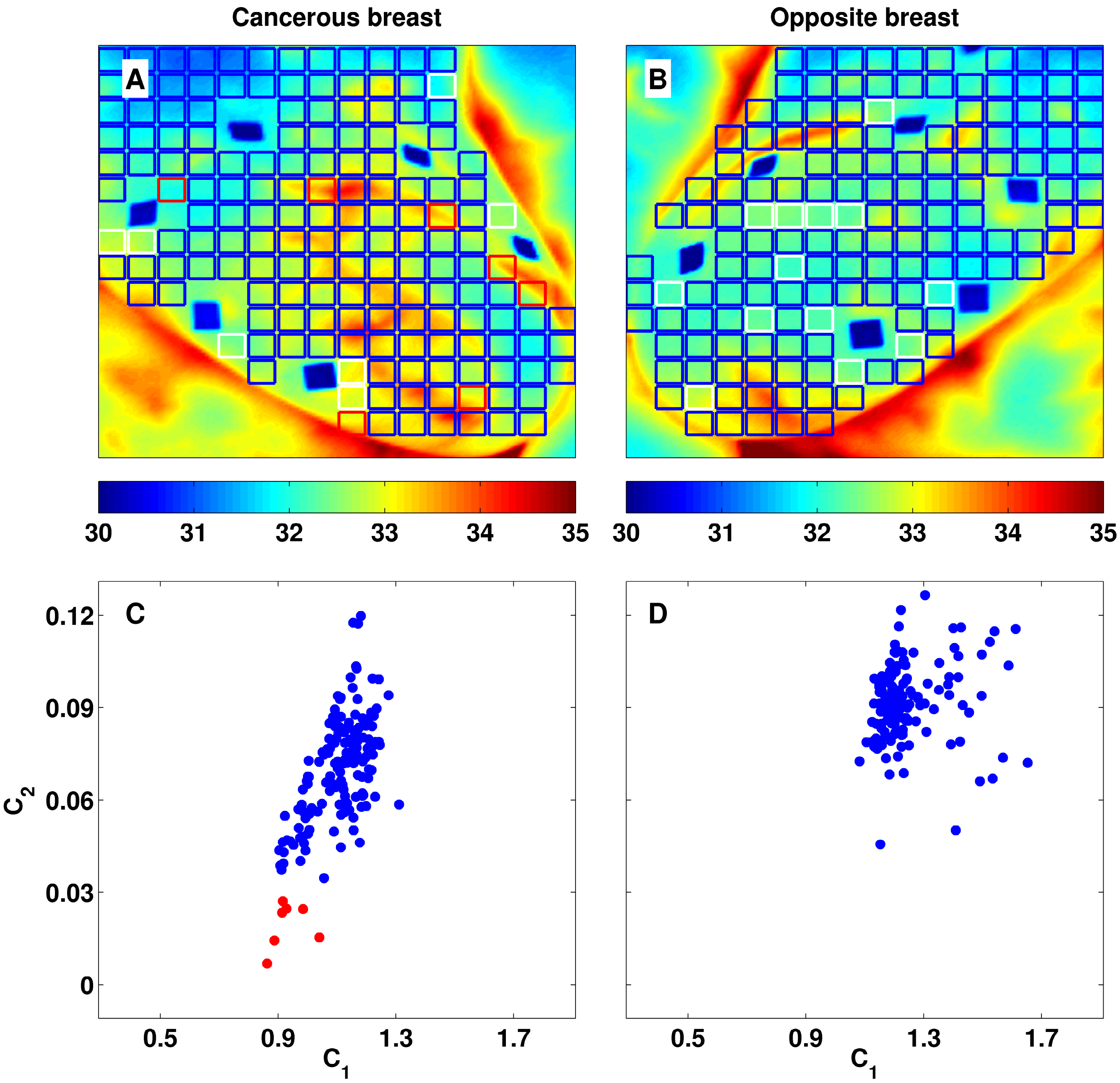

Supplementary Figure 7. Breast-wide multifractal analysis of skin (cumulative) temperature temporal fluctuations. As estimated from the $\tau(q)$ spectra computed with the WTMM method (Figs 1 and 2), 8×8 pixel^2 squares covering the two breasts of patient 27 (age 62) are color coded (A, B) and represented as a dot in the (c_1, c_2) plane (C, D). The colors have the following meaning: $c_2 < 0.03$ (red), $c_2 \geqslant 0.03$ (blue) and no scaling (white). (A, C) Cancerous right breast; (B, D) opposite left breast.

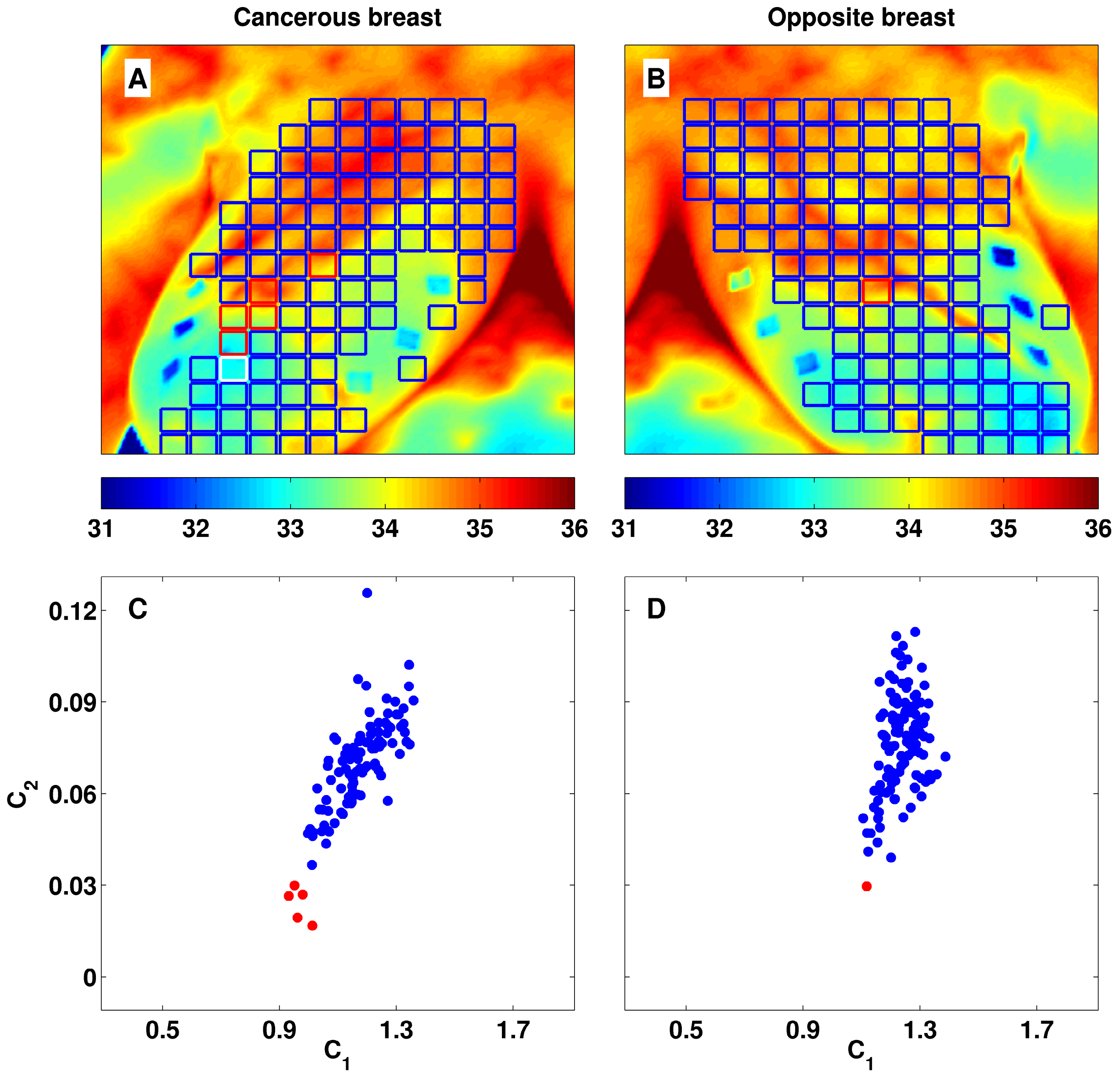

Supplementary Figure 8. Breast-wide multifractal analysis of skin (cumulative) temperature temporal fluctuations. As estimated from the $\tau(q)$ spectra computed with the WTMM method (Figs 1 and 2), 8×8 pixel^2 squares covering the two breasts of patient 12 (age 59) are color coded (A, B) and represented as a dot in the (c_1, c_2) plane (C, D). The colors have the following meaning: $c_2 < 0.03$ (red), $c_2 \geqslant 0.03$ (blue) and no scaling (white). (A, C) Cancerous right breast; (B, D) opposite left breast.

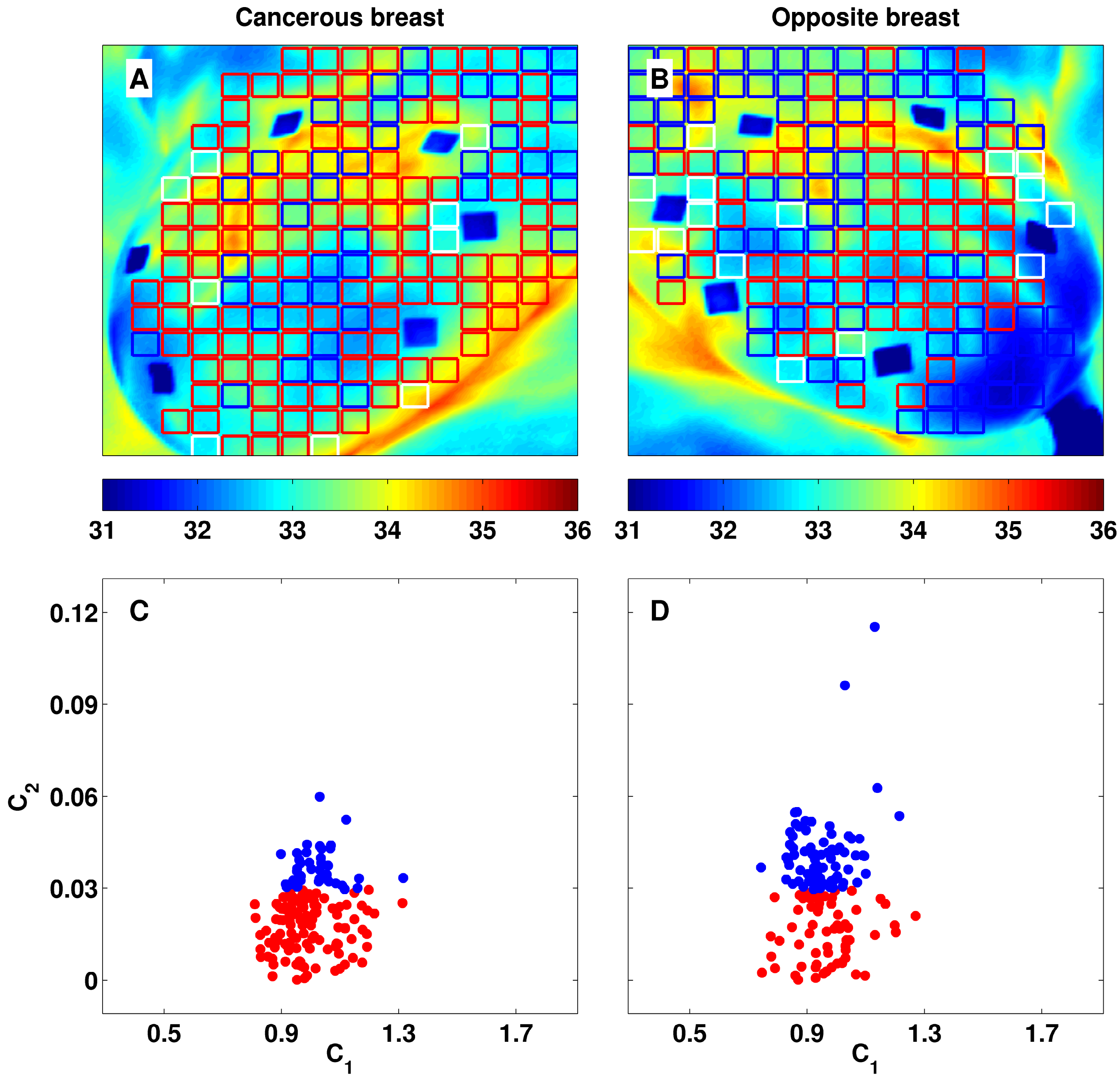

Supplementary Figure 9. Breast-wide multifractal analysis of skin (cumulative) temperature temporal fluctuations. As estimated from the $\tau(q)$ spectra computed with the WTMM method (Figs 1 and 2), 8×8 pixel^2 squares covering the two breasts of patient 30 (age 54) are color coded (A, B) and represented as a dot in the (c_1, c_2) plane (C, D). The colors have the following meaning: $c_2 < 0.03$ (red), $c_2 \geqslant 0.03$ (blue) and no scaling (white). (A, C) Cancerous right breast; (B, D) opposite left breast.

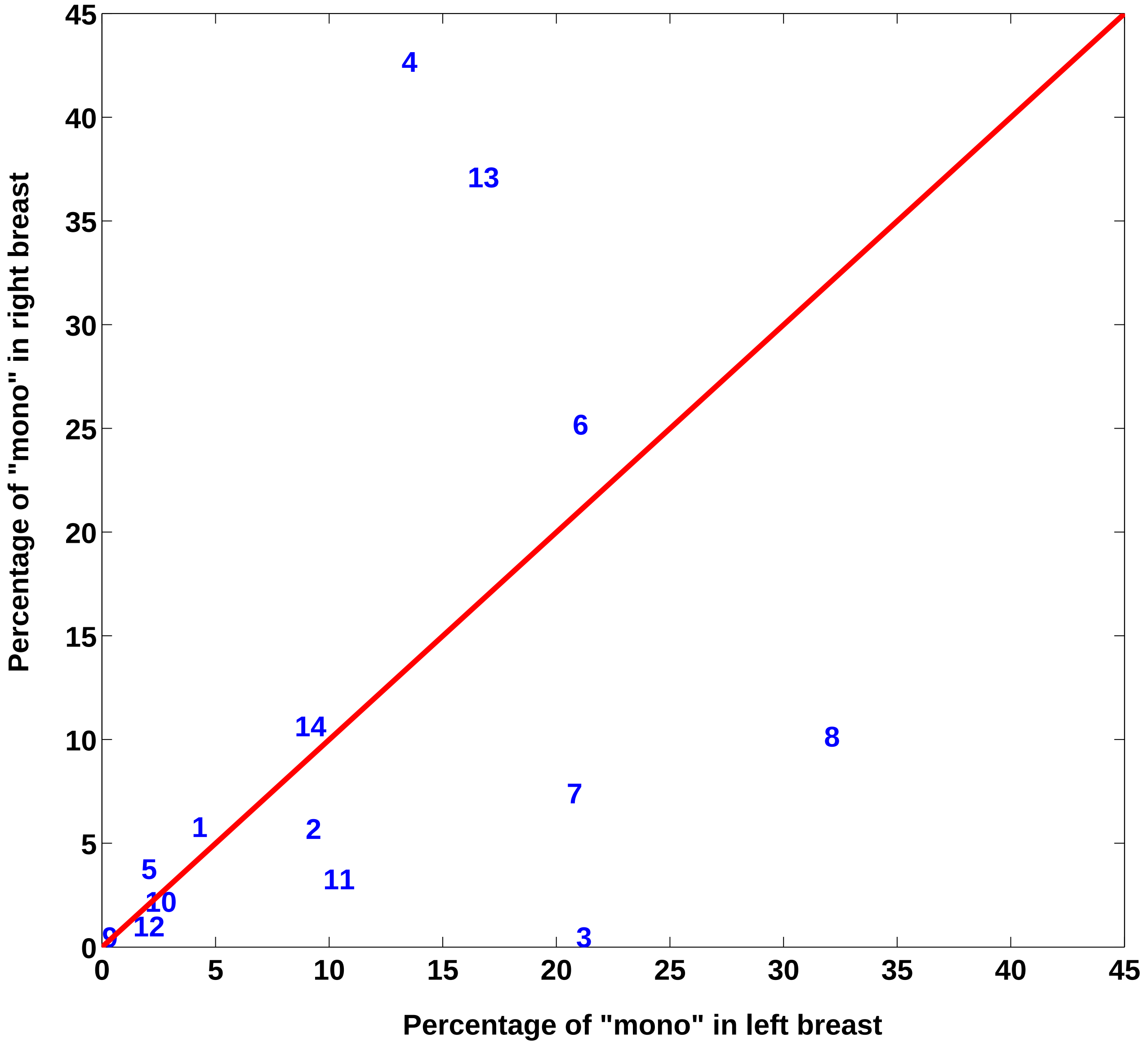

Supplementary Figure 10. Comparative analysis of the two breasts of 14 healthy volunteers. Percentage of "monofractal" 8×8 pixel^2 squares in the right breast vs the percentage in the left breast.

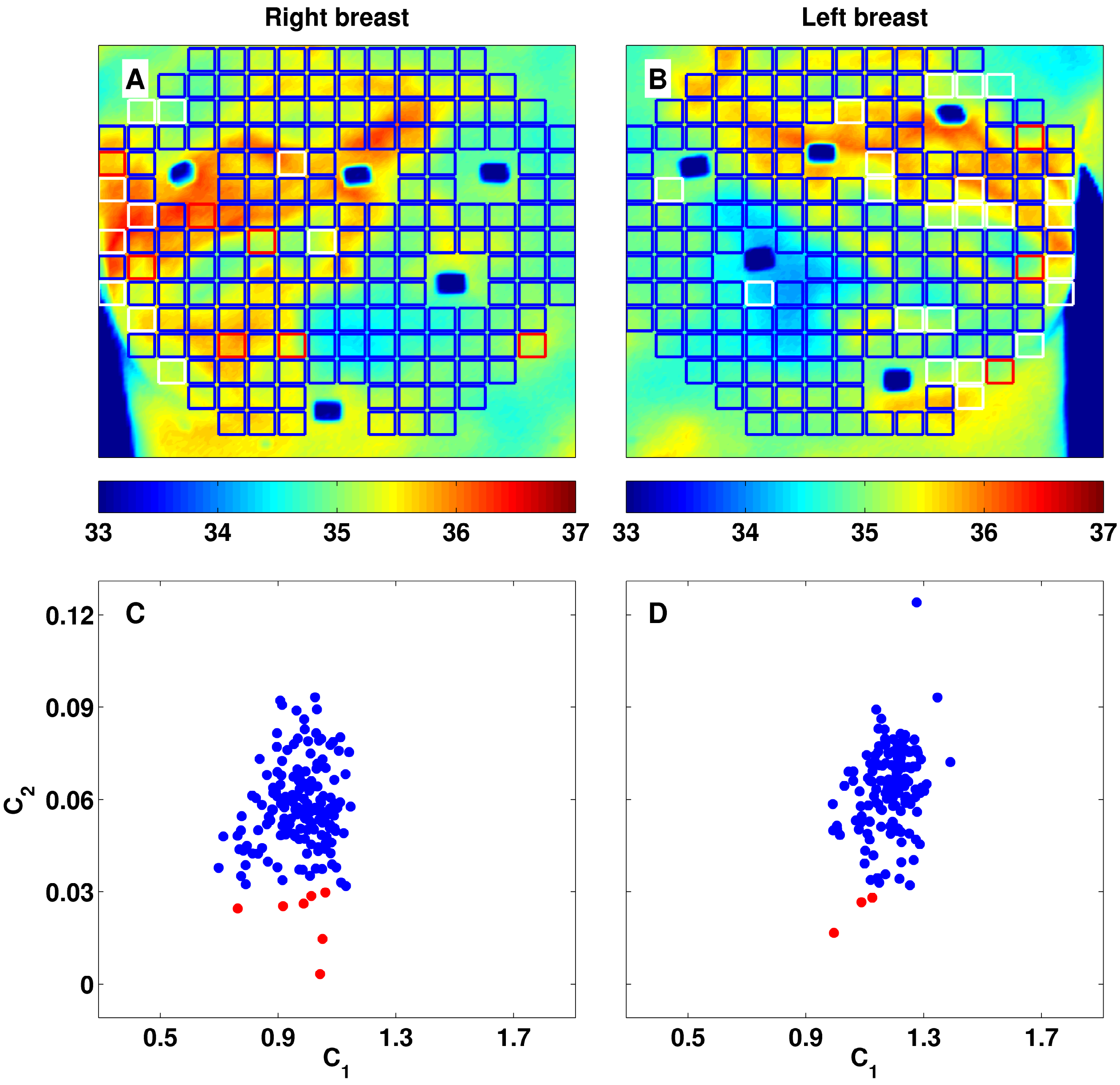

Supplementary Figure 11. Breast-wide multifractal analysis of skin (cumulative) temperature temporal fluctuations. As estimated from the $\tau(q)$ spectra computed with the WTMM method (Fig. 1 and 2), 8×8 pixel^2 squares covering the two breasts of volunteer 5 (age 26) are color coded (A, B) and represented as a dot in the (c_1, c_2) plane (C, D). The colors have the following meaning: $c_2 < 0.03$ (red), $c_2 \geqslant 0.03$ (blue) and no scaling (white). (A, C) Cancerous right breast; (B, D) opposite left breast.

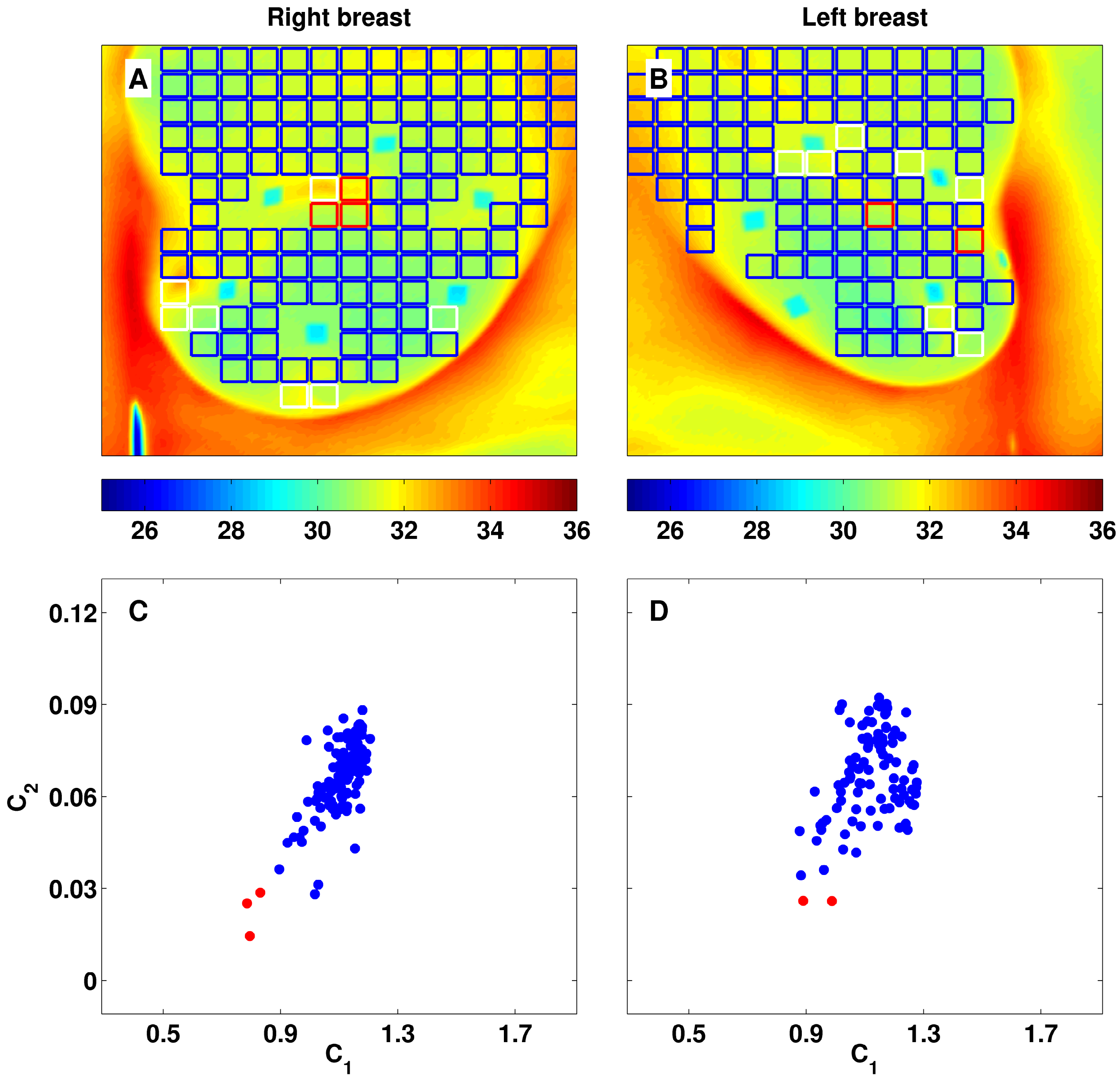

Supplementary Figure 12. Breast-wide multifractal analysis of skin (cumulative) temperature temporal fluctuations. As estimated from the $\tau(q)$ spectra computed with the WTMM method (Fig. 1 and 2), 8×8 pixel^2 squares covering the two breasts of volunteer 10 (age 55) are color coded (A, B) and represented as a dot in the (c_1, c_2) plane (C, D). The colors have the following meaning: $c_2 < 0.03$ (red), $c_2 \geqslant 0.03$ (blue) and no scaling (white). (A, C) Cancerous right breast; (B, D) opposite left breast.
